# Prenatal SARS‐CoV‐2 infection, COVID‐19 vaccination, and preschool neurodevelopment: A confounder‐adjusted cohort study

**DOI:** 10.1002/jcv2.70187

**Published:** 2026-11-09

**Authors:** Marco Rizzo, Rushna Tubassum, Floriana Milazzo, Yasemin Schmitt, Whitney Lieb, Maria De Las Mercedes Perez‐Rodriguez, Lot D. de Witte, Veerle Bergink, Anna‐Sophie Rommel

**Affiliations:** ^1^ Department of Psychiatry Icahn School of Medicine at Mount Sinai New York New York USA; ^2^ Department of Obstetrics Gynecology and Reproductive Science Icahn School of Medicine at Mount Sinai New York New York USA; ^3^ Department of Obstetrics & Gynecology Marin Health Medical Network Greenbrae California USA; ^4^ Department of Psychiatry Radboud UMC Nijmegen the Netherlands; ^5^ Department of Child and Adolescent Psychiatry/Psychology Erasmus MC University Rotterdam the Netherlands

**Keywords:** child neurodevelopment, COVID‐19 vaccination, prenatal exposure, SARS‐CoV‐2 infection

## Abstract

**Background:**

The impact of maternal SARS‐CoV‐2 infection and COVID‐19 vaccination during pregnancy on child neurodevelopment remains unclear, with most studies limited by small samples and short follow‐up periods. This prospective cohort study assesses internalizing, externalizing, total problem behaviors, and social responsiveness in children prenatally exposed or unexposed to maternal SARS‐CoV‐2 infection or COVID‐19 vaccination.

**Methods:**

The study leveraged a large NYC pregnancy cohort with detailed information on maternal infection and vaccination during pregnancy. Outcomes included parent‐reported CBCL and SRS. Analyses were adjusted for key sociodemographic and clinical factors, including race/ethnicity, maternal mental health condition, and child age at assessment.

**Results:**

The study included 675 children (mean age = 3.2 years, SD = 0.8, 50.7% male), comprising 118 children prenatally exposed to SARS‐CoV‐2 and 557 unexposed. Regression analyses showed no significant associations between prenatal SARS‐CoV‐2 exposure and CBCL Internalizing (*β* = 0.04 [95% CI: −0.85, 0.88]), CBCL Externalizing (*β* = −0.31 [95% CI: −1.41, 0.87]), CBCL Total Problems (*β* = −0.50 [95% CI: −3.46, 2.51]), and SRS (*β* = −0.60 [95% CI: −4.09, 3.17]) scores. Comparing children prenatally exposed (*n* = 68) and unexposed (*n* = 607) to COVID‐19 vaccination revealed no significant associations with CBCL Internalizing (*β* = 0.41 [95% CI: −0.52, 1.44]), CBCL Externalizing (*β* = 1.42 [95% CI: −0.32, 2.95]), CBCL Total Problems (*β* = 2.13 [95% CI: −1.22, 6.15]), and SRS (*β* = −3.65 [95% CI: −7.93, 0.32]) scores. Sociodemographic covariates, such as race/ethnicity and educational attainment, were significantly associated with all neurodevelopmental outcomes.

**Conclusion:**

Prenatal exposure to SARS‐CoV‐2 infection or COVID‐19 vaccination was not associated with adverse neurodevelopmental outcomes in preschool children. Sociodemographic factors showed stronger associations than infection or vaccination, underscoring the importance of confounder adjustment. Findings provide reassurance to pregnant individuals regarding potential long‐term neurodevelopmental outcomes in offspring following SARS‐CoV‐2 infection or COVID‐19 vaccination during pregnancy.

## Key Points

**What's known?**

- Evidence on the association between prenatal exposure to SARS‐CoV‐2 infection or COVID‐19 vaccination and child neurodevelopment has methodological limitations, including short follow‐up periods and a lack of rigorous adjustment for confounders.

**What's new?**

- To our knowledge, this study provides the longest follow‐up, through 6 years of age, and uses rigorous adjustment for key sociodemographic and clinical confounders to evaluate the associations of prenatal SARS‐CoV‐2 infection and COVID‐19 vaccination with long‐term child neurodevelopment.

**What's relevant?**

- Findings support the absence of detectable adverse neurodevelopmental associations following prenatal exposure to SARS‐CoV‐2 infection and COVID‐19 vaccination.
- Findings underscore the importance of rigorous confounder adjustment when studying prenatal exposures and child neurodevelopment.

## INTRODUCTION

The COVID‐19 pandemic exposed millions of pregnant individuals to a novel viral pathogen and a rapidly deployed vaccine, both of unknown consequences for fetal neurodevelopment. Understanding the potential risks associated with both infection and vaccine exposures requires consideration of the biological mechanisms through which they may affect the developing fetus. Infections and other inflammatory processes during pregnancy can trigger maternal immune responses, characterized by the release of inflammatory markers that may cross the placental barrier and disrupt fetal brain development during sensitive periods (Han et al., 2021; McEwan et al., 2023; Mohebalizadeh et al., 2024). Evidence from both animal models (Aria et al., 2020; Kiriyama et al., 2025) and human studies (Rasmussen et al., 2019; Suleri et al., 2022) has linked the maternal inflammatory response to altered fetal brain development and adverse neurodevelopmental outcomes in the offspring. Therefore, it has been hypothesized that SARS‐CoV‐2 infection during pregnancy could induce a maternal inflammatory response and affect fetal neurodevelopment. Although vaccination during pregnancy is generally considered safe, it induces a transient but robust systemic immune response, particularly after the first dose (Ivanova et al., 2023). When COVID‐19 vaccines were introduced, uncertainty emerged regarding the balance between the benefits of maternal vaccination and potential fetal risks. Addressing gaps in the evidence on the long‐term neurodevelopmental consequences of both exposures remains critical, as unresolved uncertainty may contribute to concerns about SARS‐CoV‐2 infection and hesitancy toward COVID‐19 vaccination observed among pregnant individuals.

The existing literature on prenatal SARS‐CoV‐2 infection and COVID‐19 vaccination is limited by methodological constraints and has not established a causal relationship between these exposures and child neurodevelopmental outcomes. In a recent systematic review of 70 studies, our team examined the associations between these prenatal exposures and child neurodevelopmental outcomes (Rizzo et al., 2025). Findings were inconsistent across studies and often did not persist with longer follow‐up. For instance, early studies based on ICD‐10 diagnostic codes suggested an elevated risk of motor and language disorders following prenatal SARS‐CoV‐2 exposure, but these associations attenuated over time (Edlow et al., 2022, 2023). Evidence of delays in personal‐social skills was largely confined to studies of severe maternal infection or to samples that conflated prenatal and postnatal infection (Garrido‐Torres et al., 2026; Jackson et al., 2024; Mulkey et al., 2022), limiting causal interpretation. In contrast, several larger controlled cohorts found no associations with early neurodevelopment (e.g., Firestein et al., 2023; Jaswa, Huddleston, et al., 2024; Vrantsidis et al., 2024).

Beyond the inconsistencies, the literature demonstrates recurring methodological weaknesses. Adverse findings came mostly from studies with small samples (*n* < 40), without an adequate control group, or with follow‐up limited to the first 2 years of life (e.g., Hill et al., 2024; Mulkey et al., 2022; Orioli et al., 2024; Purpura et al., 2023). This window may be too narrow to capture neurodevelopmental difficulties that often become apparent as children approach preschool age. Most importantly, few studies adequately accounted for sociodemographic confounding. SARS‐CoV‐2 infection during pregnancy disproportionately affected individuals with lower socioeconomic status and pre‐existing health conditions, including mental health disorders (Douglas & Subica, 2020; Janevic et al., 2022), factors that are themselves associated with adverse child neurodevelopmental outcomes (Fajardo‐Martinez et al., 2024; Jaswa, Huddleston, et al., 2024; Shuffrey et al., 2022; Vrantsidis et al., 2024; Werchan et al., 2024). Observed associations between prenatal SARS‐CoV‐2 exposure and child neurodevelopment may therefore partly reflect these underlying vulnerabilities rather than a direct biological effect of infection. Robust adjustment for these confounders is needed to isolate the contribution of prenatal SARS‐CoV‐2 infection and COVID‐19 vaccination to child neurodevelopmental outcomes. To address these methodological limitations, we leveraged a richly phenotyped pregnancy cohort with detailed exposure information and standardized neurodevelopmental child assessments at 2–6 years of age, when key cognitive, language, and behavioral domains can be reliably evaluated. Primary analyses examined associations between prenatal SARS‐CoV‐2 infection and COVID‐19 vaccination exposures and child neurodevelopmental outcomes, with comprehensive adjustment, addressing the confounding gap identified in prior work. Secondary analyses tested whether SARS‐CoV‐2 infection severity and infection in the third trimester, factors potentially linked to greater neurodevelopmental risk (Hall et al., 2023; Hardie, Murray, et al., 2025; Suleri et al., 2024), were associated with poorer outcomes among exposed children.

## METHODS

### Protocol pre‐registration

The study was pre‐registered on Open Science Framework in January 2026. The design and analysis plan are available at: https://osf.io/n7rqw/overview?view_only=66e4dcee056f4d23819dd7f05d0075f2.

### Deviations from protocol pre‐registration

Two covariates were added beyond the pre‐registered analysis plan. Parity (nulliparous and multiparous) was added as a confounder given evidence from our prior work showing that multiparous women were significantly more likely to have contracted SARS‐CoV‐2 during pregnancy (Gigase et al., 2024), alongside well‐documented associations between birth order and child neurodevelopment (Tsuchida et al., 2026). Pandemic timing, operationalized as days elapsed between March 1, 2020 (the date of New York City's first confirmed COVID‐19 case) and the child's date of birth, was included as a covariate to account for variation in prenatal environmental conditions, healthcare access, and pandemic‐related stressors that may influence neurodevelopmental outcomes independently of direct prenatal SARS‐CoV‐2 exposure. Furthermore, although family‐level clustering of standard errors was pre‐registered, only three sibling pairs were present in the analytic sample. Accordingly, one sibling from each pair was randomly excluded and analyses were conducted on independent observations. Lastly, two post hoc sensitivity analyses were conducted, restricting the sample to participants recruited through the original Gen C cohort and stratifying by child age at assessment (Section 2.8.3).

### Study population

Participants (*N* = 675) were recruited at Mount Sinai Health System (MSHS) in New York City through the Generation C (Gen C; 2020–2022) cohort (Gigase et al., 2024) and its extension, Generation C‐SF (Gen C‐SF; 2022‐present), which continued enrollment within the same institutional setting. The present analysis included a subset of mother–child dyads participating in the ongoing child follow‐up study, with children assessed between November 2022 and October 2026 at a median age of 3.3 (IQR: 2.6–3.8) years. All children were born between April 2020 and July 2023, most in 2020 (*n* = 370, 54.8%) or 2021 (*n* = 193, 28.6%), with fewer in 2022 (*n* = 97, 14.4%) and 2023 (*n* = 15, 2.2%). A distribution of the birth dates for the children in the included sample is reported in Supporting Information S1: Appendix S1. All participants provided informed consent.

### Exposure definition

Maternal SARS‐CoV‐2 infection status was ascertained through (1) a positive RT‐PCR test result during pregnancy, with the date of the positive test considered the date of SARS‐CoV‐2 detection; (2) evidence of prior infection based on anti‐spike (anti‐S) IgG antibody positivity in conjunction with one of the following: (a) anti‐S IgG positivity before an individual's first COVID‐19 vaccination, (b) anti‐S IgG positivity before the COVID‐19 vaccination rollout in NYC (December 14, 2020), or (c) concurrent anti‐S IgG and anti‐nucleocapsid (anti‐N) IgG positivity; or (3) a diagnosis documented by a healthcare provider in the electronic medical record (EMR) and/or reported on a study questionnaire during pregnancy, with the date of documentation considered the date of SARS‐CoV‐2 detection. Participants were classified as never infected if no evidence of SARS‐CoV‐2 infection was identified using any of the criteria described above (Gigase et al., 2024). Because serology alone cannot establish a precise infection date, exposure timing for serology‐based cases was assigned at the level of trimester. For participants with serial samples, the interval between the last negative and first positive sample was mapped onto pregnancy dating (last menstrual period, trimester boundaries, and delivery date) to assign trimester of infection. Where dated EMR results or self‐reported timing were consistent with the serologic interval, they were used to refine placement, allowing assignment in participants whose sampling interval alone spanned more than one trimester. Serology‐derived timing was then integrated with evidence from positive PCR testing and chart‐documented infection, with the earliest trimester across modalities defining timing of first infection.

Infection severity was classified based on self‐report and EMR data according to World Health Organization criteria (World Health Organization, 2020) as: (a) asymptomatic/presymptomatic (i.e., no detectable symptoms); (b) mild (e.g., fever, cough, fatigue); (c) moderate (e.g., difficulty breathing or mild pneumonia); and (d) severe (e.g., severe or life‐threatening illness). Because only one participant met criteria for severe infection, the moderate and severe categories were combined for the analyses.

Information on prenatal COVID‐19 vaccination status was obtained through self‐report and from the maternal EMR, which is linked to the New York Citywide Immunization Registry (CIR). The CIR consolidates all immunization information across NYC, including vaccination outside of the MSHS (e.g., pharmacies). Reporting of all COVID‐19 vaccine doses to the CIR is required within 24 h of administration. Participants were classified as vaccinated if they received at least one dose of the Janssen (Johnson and Johnson) (Ad26.COV2.S) viral‐vector vaccine or one of the mRNA vaccines, Moderna (mRNA‐1273) or Pfizer‐BioNTech (BNT162b2), at any point during pregnancy. No participants reported receiving a COVID‐19 vaccine not authorized in the US. Timing of vaccination (before, during, or after pregnancy) and trimester of each dose were determined using gestational age established by ultrasounds (Ibroci et al., 2023).

### Outcomes

Children's behavioral and social functioning were assessed using two validated parent‐report instruments. The preschool Child Behavior Checklist (CBCL 1½‐5) (Achenbach & Rescorla, 2000) assesses behavioral and emotional problems across 99 items rated on a 3‐point Likert scale (0 = “Not true” to 2 = “Very often or often true”), reflecting the child's behavior over the preceding two months. Items load onto seven syndrome subscales, from which two broadband scales are derived: Internalizing (Emotionally Reactive, Anxious/Depressed, Somatic Complaints, and Withdrawn; 36 items) and Externalizing (Attention Problems and Aggressive Behaviors; 24 items). A Total Problems score is computed from all 99 items. Higher scores indicate greater behavioral and emotional problems. T‐scores below 60 are considered within the typical range. Raw scores were used in all analyses.

The Social Responsiveness Scale, Second Edition (SRS‐2) Preschool Form (Constantino & Gruber, 2012) assesses social communication difficulties and restricted or repetitive behaviors across 65 items rated on a 4‐point Likert scale (1 = “Not true” to 4 = “Almost always true”). The SRS comprises five subscales: Social Awareness, Social Cognition, Social Communication, Social Motivation, and Restricted Interests and Repetitive Behavior. Higher scores indicate greater social impairment. T‐scores below 65 are considered within the typical range. Total raw scores were used in all analyses.

When multiple assessments were available for a given child (*n* = 37 for CBCL and *n* = 29 for SRS), the most recent observation was retained; scores were strongly correlated between the first and second assessments (Supporting Information S1: Appendix S2; Figure S1; Table S1). Three children were assessed shortly after their sixth birthday (maximum age 6 years 3 months), beyond the age covered by the CBCL 1 ½–5 and the SRS‐2 Preschool Form designed for children aged 2.5–4.5 years. Children assessed outside this range were retained in the analyses.

### Covariates

Covariates were selected based on prior literature and previous findings from this cohort (Gigase et al., 2024; Ibroci et al., 2023; Molenaar et al., 2022). Relationships among exposures, outcomes, and covariates were formalized using directed acyclic graphs (DAGs; Supporting Information S1: Appendix S3).

For all analyses, covariates included maternal age at delivery (years; continuous), race/ethnicity (Asian, Black, Hispanic, White, or Other; categorical), educational attainment (no high school diploma, high school/some college, or higher education; categorical), parity (nulliparous, multiparous; categorical), pre‐pregnancy body mass index (BMI, kg/m^2^; continuous), history of maternal mental illness (yes/no; categorical), gestational age at birth (days; continuous), pandemic timing (defined as the number of days between March 1, 2020, and the child's date of birth; continuous), child sex (female/male; categorical), and child age at assessment (months; continuous).

### Inclusion and exclusion criteria

Eligible participants were mother–child dyads enrolled in the Generation C follow‐up study in which the mother was aged 18 years or older at initial enrollment, received obstetrical care at MSHS, and provided informed consent. Dyads were excluded if they met *any* of the following exclusion criteria: (i) unknown maternal SARS‐CoV‐2 infection status during pregnancy; (ii) multiple gestation pregnancy (e.g., twins, triplets); or missing outcome data per published instrument guidelines, defined as (iii) more than 8 missing items on the CBCL or (iv) more than 10% missing items on the SRS. Exclusion due to missing outcome data was applied independently for each instrument. Accordingly, dyads with insufficient data for one outcome remained eligible for analyses involving the other outcome measure. A flowchart of excluded participants is reported in Supporting Information S1: Appendix S4.

### Statistical analysis

All analyses were conducted in R 4.3.1.

#### Research questions

This study addressed four research questions. The primary analyses tested whether prenatal exposure to (i) SARS‐CoV‐2 infection or (ii) COVID‐19 vaccination was associated with child neurodevelopmental outcomes, including CBCL Internalizing, Externalizing, Total Problems, and Social Responsiveness Scale scores. Secondary analyses were restricted to the SARS‐CoV‐2 infection exposure group and examined whether (iii) higher infection severity or (iv) third‐trimester infection were associated with neurodevelopmental outcomes across the same four measures.

#### Descriptive statistics

Group differences in dyad characteristics were assessed using Pearson's Chi‐square test for categorical variables and Wilcoxon rank‐sum test for continuous variables. Tables 1 and 2 present descriptive statistics for neurodevelopmental outcomes, exposures, and dyad characteristics in the overall sample and by SARS‐CoV‐2 infection and COVID‐19 vaccination exposure status, respectively. Supporting Information S1: Appendices S5 and S6 provide analogous descriptive statistics stratified by infection severity and gestational timing of infection, respectively. CBCL and SRS score distributions relative to clinical cutoffs and the prevalence of children exceeding those thresholds are reported in Supporting Information S1: Appendices S7 and S8.

**TABLE 1:** Neurodevelopmental survey scores and cohort characteristics stratified by prenatal SARS‐CoV‐2 infection exposure.

Characteristic	Overall	SARS‐CoV‐2 exposed	SARS‐CoV‐2 unexposed	*p*‐value^a^
	*N* = 675	*n* = 118	*n* = 557	
CBCL—Internalizing				0.28
Median (Q1, Q3)	3.0 (1.0, 6.0)	3.0 (1.0, 7.8)	2.0 (1.0, 6.0)	
CBCL—Externalizing				0.43
Median (Q1, Q3)	4.0 (1.0, 9.0)	4.0 (1.0, 10.8)	4.0 (1.0, 9.0)	
CBCL—Total problems				0.41
Median (Q1, Q3)	11.0 (5.0, 23.5)	13.0 (5.0, 26.0)	11.0 (5.0, 23.0)	
SRS raw score				0.25
Median (Q1, Q3)	28.0 (18.0, 41.0)	29.0 (18.5, 46.0)	28.0 (18.0, 40.0)	
Unexposed = 548				
Exposed = 114				
Trimester of exposure^b^, *n* (%)				‐
First trimester	NA	17 (14.4%)	NA	
Second trimester		35 (29.7%)		
Third trimester		66 (55.9%)		
Disease severity^b^, *n* (%)				‐
Asymptomatic	NA	60 (50.8%)	NA	
Mild		37 (31.4%)		
Moderate/Severe		18 (15.3%)		
Missing		3 (2.5%)		
Vaccine exposure^b^, *n* (%)				0.07
Exposed	68 (10.1%)	6 (5.1%)	62 (11.1%)	
Unexposed	607 (89.9%)	112 (94.9%)	495 (88.9%)	
Pandemic timing (days)				**0.02**
Median (Q1, Q3)	285.0 (157.0, 527.0)	318.5 (171.0, 839.5)	285.0 (155.0, 472.0)	
Maternal age at delivery (years)				0.19
Median (Q1, Q3)	33.0 (30.0, 36.0)	32.5 (30.0, 36.0)	34.0 (30.0, 37.0)	
Race/Ethnicity, *n* (%)				**<0.001**
Asian	79 (11.7%)	5 (4.2%)	74 (13.3%)	
Black	116 (17.2%)	29 (24.6%)	87 (15.6%)	
Hispanic	178 (26.4%)	45 (38.1%)	133 (23.9%)	
White	293 (43.4%)	36 (30.5%)	257 (46.1%)	
Other	9 (1.3%)	3 (2.5%)	6 (1.1%)	
Educational attainment, *n* (%)				0.28
No high school diploma	30 (4.4%)	7 (5.9%)	23 (4.1%)	
High school/Some college	181 (26.8%)	37 (31.4%)	144 (25.9%)	
College degree or above	464 (68.7%)	74 (62.7%)	390 (70.0%)	
Maternal pre‐pregnancy BMI (kg/m^2^)				**<0.001**
Median (Q1, Q3)	25.7 (22.3, 30.7)	28.9 (24.2, 33.2)	25.2 (22.0, 30.2)	
Parity, *n* (%)				**<0.01**
Nulliparous	338 (50.1%)	44 (37.3%)	294 (52.8%)	
Multiparous	337 (49.9%)	74 (62.7%)	263 (47.2%)	
History of maternal mental illness, *n* (%)				**<0.01**
Yes	262 (38.8%)	59 (50.0%)	203 (36.4%)	
No	413 (61.2%)	59 (50.0%)	354 (63.6%)	
Child sex, *n* (%)				0.39
Female	333 (49.3%)	63 (53.4%)	270 (48.5%)	
Male	342 (50.7%)	55 (46.6%)	287 (51.5%)	
Gestational age at birth (days)				0.45
Median (Q1, Q3)	274.0 (267.0, 279.0)	274.0 (264.0, 278.0)	274.0 (268.0, 279.0)	
Age at assessment (years)				**0.03**
Median (Q1, Q3)	3.3 (2.6, 3.8)	3.3 (3.1, 3.8)	3.2 (2.5, 3.8)	
Recruitment cohort, *n* (%)				**<0.001**
Gen C	634 (93.9%)	101 (85.6%)	533 (95.7%)	
Gen C‐SF	41 (6.1%)	17 (14.4%)	24 (4.3%)	
Child ASD/ADHD diagnosis, *n* (%)				0.22
No	627 (92.9%)	106 (89.8%)	521 (93.5%)	
Yes	48 (7.1%)	12 (10.2%)	36 (6.5%)	

Abbreviations: ADHD, attention‐deficit/hyperactivity disorder; ASD, autism spectrum disorder; BMI, body mass index; CBCL, Child Behavior Checklist; NA, not applicable; SRS, Social Responsiveness Scale.

^a^ Wilcoxon rank‐sum test; Pearson's Chi‐squared test. Significant *p*‐values appear in bold.

^b^ Data on infection severity, infection trimester, and COVID‐19 vaccination status by SARS‐CoV‐2 infection exposure are presented for descriptive purposes.

**TABLE 2:** Neurodevelopmental survey scores and cohort characteristics stratified by prenatal COVID‐19 vaccination exposure.

Characteristic	Overall	Vaccine exposed	Vaccine unexposed	*p*‐value^a^
	*N* = 675	*n* = 68	*n* = 607	
CBCL—Internalizing				0.14
Median (Q1, Q3)	3.0 (1.0, 6.0)	3.0 (1.0, 6.2)	2.0 (1.0, 6.0)	
CBCL—Externalizing				**0.049** **^b^**
Median (Q1, Q3)	4.0 (1.0, 9.0)	5.5 (2.0, 11.0)	4.0 (1.0, 9.0)	
CBCL—Total problems				**0.04** **^b^**
Median (Q1, Q3)	11.0 (5.0, 23.5)	16.0 (7.0, 24.5)	11.0 (5.0, 23.0)	
SRS raw score				**0.02** **^b^**
Median (Q1, Q3)	28.0 (18.0, 41.0)	24.5 (16.8, 34.0)	29.0 (18.0, 42.0)	
Unexposed = 594				
Exposed = 68				
Vaccine trimester, *n* (%)				‐
First trimester	NA	14 (20.6%)	NA	
Second trimester		26 (38.2%)		
Third trimester		24 (35.3%)		
Missing		4 (5.9%)		
Pandemic timing (days)				**<0.001**
Median (Q1, Q3)	285.0 (157.0, 527.0)	509.5 (421.8, 637.5)	264.0 (150.5, 450.0)	
Maternal age at delivery (years)				**<0.001**
Median (Q1, Q3)	33.0 (30.0, 36.0)	36.0 (32.0, 38.0)	33.0 (30.0, 36.0)	
Race/Ethnicity, *n* (%)				**<0.001**
Asian	79 (11.7%)	17 (25.0%)	62 (10.2%)	
Black	116 (17.2%)	5 (7.4%)	111 (18.3%)	
Hispanic	178 (26.4%)	8 (11.8%)	170 (28.0%)	
White	293 (43.4%)	37 (54.4%)	256 (42.2%)	
Other	9 (1.3%)	1 (1.5%)	8 (1.3%)	
Educational attainment, *n* (%)				**<0.001**
No high school diploma	30 (4.4%)	0 (0.0%)	30 (4.9%)	
High school/Some college	181 (26.8%)	7 (10.3%)	174 (28.7%)	
College degree or above	464 (68.7%)	61 (89.7%)	403 (66.4%)	
Maternal pre‐pregnancy BMI (kg/m^2^)				**0.03**
Median (Q1, Q3)	25.7 (22.3, 30.7)	23.7 (21.7, 27.7)	25.8 (22.4, 31.2)	
Parity, *n* (%)				0.53
Nulliparous	338 (50.1%)	37 (54.4%)	301 (49.6%)	
Multiparous	337 (49.9%)	31 (45.6%)	306 (50.4%)	
History of maternal mental illness, *n* (%)				0.77
Yes	262 (38.8%)	28 (41.2%)	234 (38.6%)	
No	413 (61.2%)	40 (58.8%)	373 (61.4%)	
Child sex, *n* (%)				0.30
Female	333 (49.3%)	29 (42.6%)	304 (50.1%)	
Male	342 (50.7%)	39 (57.4%)	303 (49.9%)	
Gestational age at birth (days)				**0.03**
Median (Q1, Q3)	274.0 (267.0, 279.0)	275.5 (271.0, 281.2)	274.0 (267.0, 278.0)	
Age at assessment (years)				**<0.001**
Median (Q1, Q3)	3.3 (2.6, 3.8)	3.5 (3.2, 4.0)	3.2 (2.6, 3.8)	
Recruitment cohort, *n* (%)				**<0.001**
Gen C	634 (93.9%)	55 (80.9%)	579 (95.4%)	
Gen C‐SF	41 (6.1%)	13 (19.1%)	28 (4.6%)	
Child ASD/ADHD diagnosis, *n* (%)				0.10
No	627 (92.9%)	67 (98.5%)	560 (92.3%)	
Yes	48 (7.1%)	1 (1.5%)	47 (7.7%)	

*Note*: Table reporting sociodemographic and clinical characteristics from mothers and children stratified by COVID‐19 vaccination exposure status.

Abbreviations: ADHD, attention‐deficit/hyperactivity disorder; ASD, autism spectrum disorder; BMI, body mass index; CBCL, Child Behavior Checklist; NA, not applicable; SRS, Social Responsiveness Scale.

^a^ Wilcoxon rank‐sum test; Pearson's Chi‐squared test. Significant *p*‐values appear in bold.

^b^ Unadjusted *p*‐value. Vaccine‐exposed children had higher CBCL Externalizing and Total Problems scores and lower (i.e., more favorable) SRS scores than unexposed children; none of these differences was statistically significant after covariate adjustment (Figure 1; Supporting Information S1: Appendices S18–S21).

#### Regression models

For each exposure (SARS‐CoV‐2 infection and COVID‐19 vaccination), separate multivariable linear regression models were fitted for the four neurodevelopmental outcomes. Outcome skewness was assessed using Fisher‐Pearson coefficient (Supporting Information S1: Appendix S9). When skewness exceeded 2, robust linear regression with heteroscedasticity‐consistent standard errors was employed. For each analysis, minimally and fully adjusted models were estimated. Minimally adjusted models included maternal age at delivery, race/ethnicity, educational attainment, parity, pandemic timing, child sex, and child age at assessment. Fully adjusted models additionally included pre‐pregnancy BMI, history of maternal mental illness, and gestational age at birth. Results from minimally adjusted models only are reported in the Supporting Information file as they provided no additional substantive insights beyond the fully adjusted specification. False Discovery Rate correction was applied within each research question across the four outcomes.

For the primary analysis, maternal SARS‐CoV‐2 infection was coded as binary (exposed vs. unexposed). Prenatal COVID‐19 vaccination was also examined as a binary exposure (vaccinated vs. unvaccinated), with participants classified as vaccinated if they received at least one COVID‐19 vaccine dose during pregnancy. Individuals vaccinated exclusively before pregnancy were classified as unvaccinated under the assumption that transient vaccine‐induced inflammatory responses would not extend into the gestational period and would therefore be unlikely to affect fetal neurodevelopment through the hypothesized mechanism of transient maternal immune response. To test the robustness of this classification decision, a sensitivity analysis was conducted excluding individuals vaccinated prior to pregnancy from the unvaccinated reference group. Due to the limited number of participants vaccinated during pregnancy (*n* = 68), analyses were not stratified by trimester. In addition, a sensitivity analysis was restricted to participants recruited through the original Gen C cohort (i.e., excluding Gen C‐SF participants, *n* = 41) to account for possible structural and procedural differences between the two study protocols. Lastly, to examine whether associations differed by age at assessment, the primary analyses were repeated within age groups (24–36, 37–48, and ≥ 49 months for SARS‐CoV‐2 infection; 24–48 and ≥ 49 months for COVID‐19 vaccination, because only seven children assessed at 24–36 months were vaccine‐exposed), and exposure × age‐group interactions were tested in the full sample.

#### Missing data

Missing covariate data were handled using multiple imputation by chained equations (MICE) (van Buuren & Groothuis‐Oudshoorn, 2011) with variable‐specific conditional models. Continuous variables were imputed using Predictive Mean Matching (‘pmm’) model, binary variables using logistic regression (‘logreg’), and categorical variables using multinomial logistic regression (‘polyreg’).

Item‐level missingness was minimal: 42 CBCL items were missing across 37 children (no child was missing more than two items), and 23 SRS‐2 items were missing across 23 children (one item each). A hybrid approach combining instrument‐specific scoring rules with multiple imputation was employed to address missing item‐level data from the SRS and CBCL (Wu et al., 2022). First, following published guidelines for each instrument, provisional scores were calculated to serve as auxiliary variables. Specifically, for the CBCL, subscales were provisionally calculated with missing items left blank (Achenbach & Rescorla, 2000). For the SRS, missing items were provisionally coded using the item‐level median value specified in the manual (Constantino & Gruber, 2012). Second, these provisional scores, alongside sociodemographic variables used in the statistical analysis, were included as auxiliary predictors in MICE using Predictive Mean Matching (‘pmm’). By integrating instrument‐based scoring rules with model‐based imputation, this two‐step approach balances the normative psychometric structure of the questionnaires with statistical efficiency, shown to yield more accurate parameter estimates and standard errors than multiple imputation or standard scoring rule alone (Wu et al., 2022).

## RESULTS

### Cohort characteristics

#### SARS‐CoV‐2 infection exposure

The final analytical sample comprised 675 children, of whom 118 (17.5%) were prenatally exposed to SARS‐CoV‐2 and 557 (82.5%) were unexposed. Complete CBCL data were available for all 675 children; SRS data were available for 662 children, of whom 114 (17.2%) were exposed. Cohort characteristics are presented in Table 1.

Exposed and unexposed groups differed across several characteristics: mothers infected with SARS‐CoV‐2 during pregnancy were more likely to identify as Hispanic or Black, to have higher pre‐pregnancy BMI, to be multiparous, to have a history of mental illness, to have older children at assessment, to have delivered later in the pandemic, and to be recruited from the Gen C‐SF cohort (Table 1).

Most infections were asymptomatic (*n* = 60, 50.8%), with 31.4% (*n* = 37) mild and 15.3% (*n* = 18) moderate‐to‐severe; severity data were missing for 3 participants (2.5%) (Table 1; Supporting Information S1: Appendix S5). Maternal SARS‐CoV‐2 infection most commonly occurred in the third trimester (*n* = 66; 55.9%), followed by the second (*n* = 35; 29.7%) and first (*n* = 17; 14.4%) trimester (Table 1; Supporting Information S1: Appendix S6).

#### COVID‐19 vaccination exposure

Of the 675 children, 68 (10.1%) were exposed to maternal COVID‐19 vaccination during pregnancy and 607 (89.9%) were unexposed. Vaccinated mothers were more likely to be older, identify as White or Asian, to have higher educational attainment, to have lower pre‐pregnancy BMI, to have older children at assessment and longer gestational age at birth, to be recruited from the Gen C‐SF cohort, and to have delivered later in the pandemic. Cohort characteristics stratified by COVID‐19 vaccination exposure are presented in Table 2.

#### Neurodevelopmental outcome distributions

Median (IQR) scores in the overall sample were 3.0 (1.0, 6.0) for CBCL Internalizing, 4.0 (1.0, 9.0) for CBCL Externalizing, 11.0 (5.0, 23.5) for CBCL Total Problems, and 28.0 (18.0, 41.0) for SRS. Most children scored within the typical range on each outcome (CBCL Internalizing: 93.0%, CBCL Externalizing: 95.1%, CBCL Total Problems: 94.5%; SRS: 93.4%) (Figures 2 and 3). Median scores by exposure status are reported in Tables 1 and 2, and full score distributions relative to the clinical cutoffs are shown in Figures 2 and 3. The number and proportion of children scoring in the borderline or clinical range, by SARS‐CoV‐2 exposure status, are reported in Supporting Information S1: Appendices S7 and S8.

**FIGURE 1:**
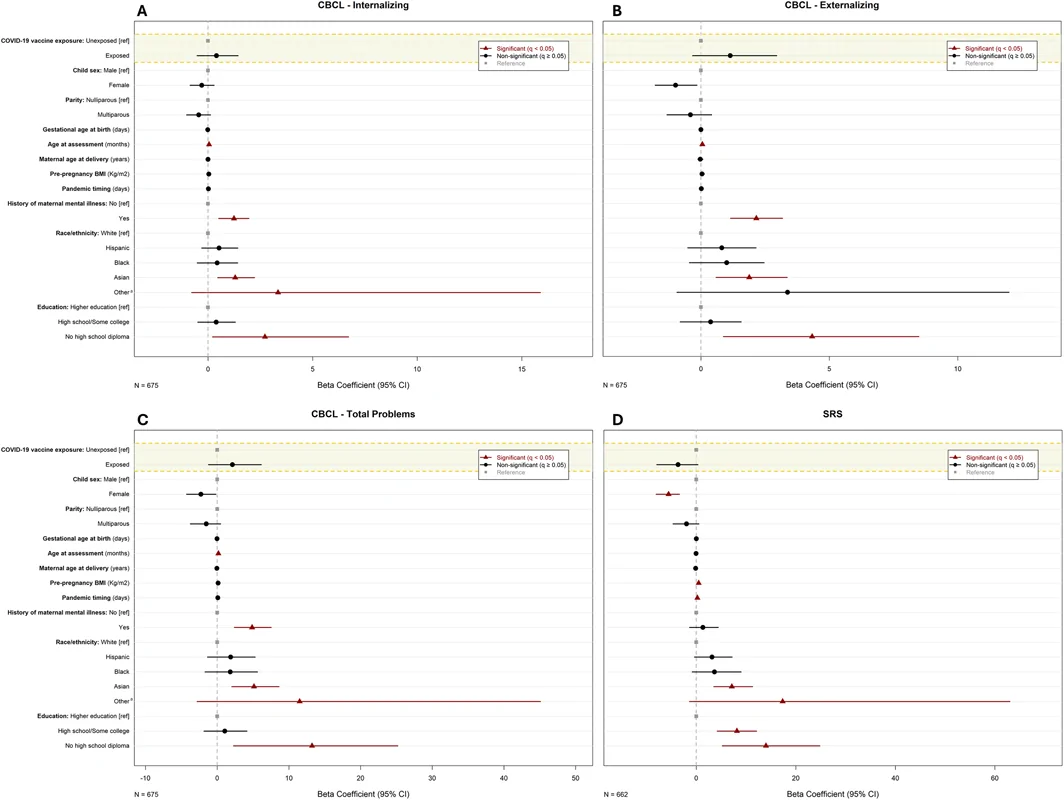
Association between prenatal exposure to COVID‐19 vaccination and neurodevelopmental outcomes with covariates. Forest plots displaying Beta coefficients with 95% confidence intervals from multivariable robust regression models examining the associations between prenatal COVID‐19 vaccination exposure and each neurodevelopmental outcome: CBCL—Internalizing (A), CBCL—Externalizing (B), CBCL—Total Problems (C), and SRS (D). The shaded area highlights the main exposure (COVID‐19 vaccination); all the other covariates included in the analysis are listed below. Reference categories correspond to the largest groups. Positive values indicate higher scores. ^a^Due to the small sample size (*n* = 9) the Race/ethnicity: Other category yielded an unstable and asymmetrical estimate. BMI, body mass index; CBCL, Child Behavior Checklist; SRS, Social Responsiveness Scale.

**FIGURE 2:**
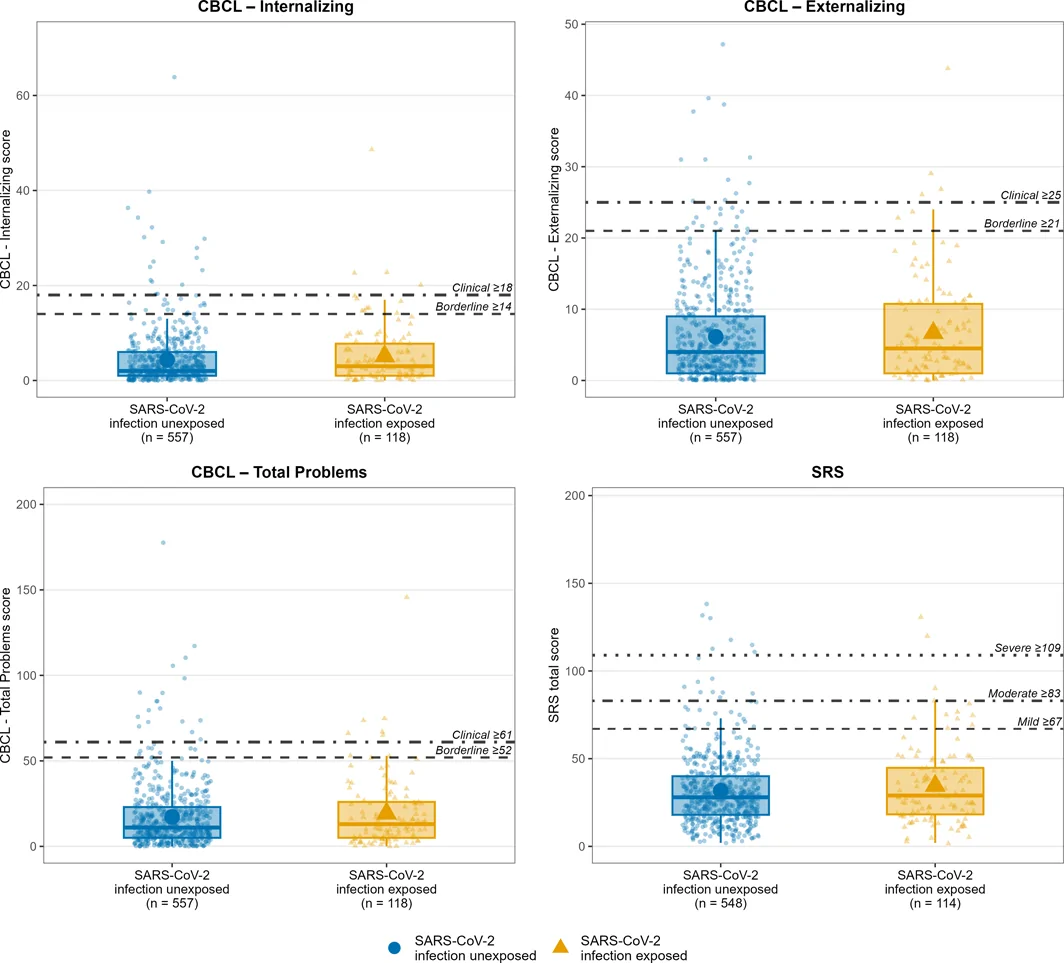
Score distribution for SRS and CBCL subscales stratified by prenatal SARS‐CoV‐2 exposure status. Boxplots showing score distribution (median and interquartile range) of neurodevelopmental outcomes from children prenatally exposed (yellow) and unexposed (blue) to SARS‐CoV‐2 infection for each neurodevelopmental outcome: CBCL—Internalizing (top left), CBCL—Externalizing (top right), CBCL—Total Problems (bottom left), and SRS (bottom right). The plots also report individual data points (yellow/blue jitter) and mean value (yellow diamond/blue circle). Dashed lines indicate clinical thresholds. *Y*‐axes span the full score range for each instrument. CBCL, Child Behavior Checklist; SRS, Social Responsiveness Scale.

**FIGURE 3:**
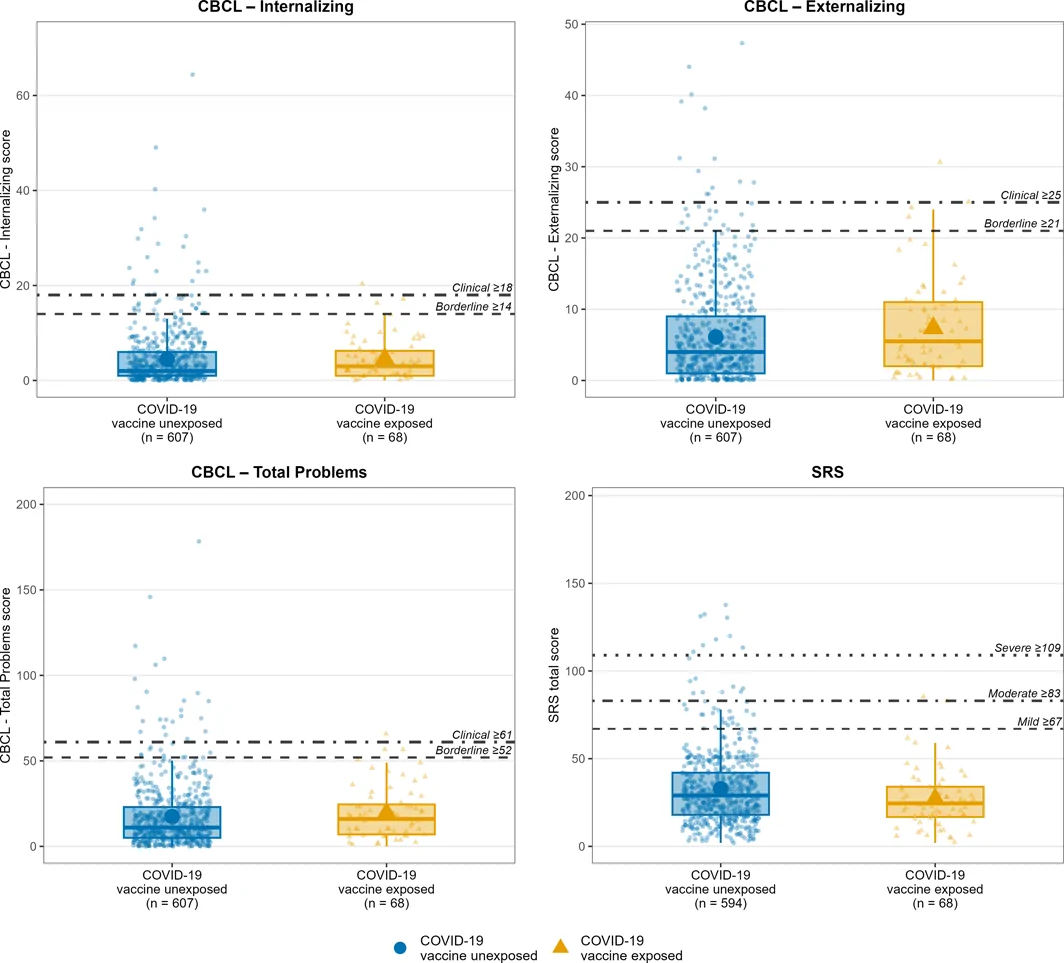
Score distribution for SRS and CBCL subscales stratified by COVID‐19 vaccination exposure status. Boxplots showing score distribution (median and interquartile range) of neurodevelopmental outcomes from children prenatally exposed (yellow) and unexposed (blue) to COVID‐19 vaccination for each neurodevelopmental outcome: CBCL—Internalizing (top left), CBCL—Externalizing (top right), CBCL—Total Problems (bottom left), and SRS (bottom right). The plots also report individual data points (yellow/blue jitter) and mean value (yellow diamond/blue circle). Dashed lines indicate clinical thresholds. *Y*‐axes span the full score range for each instrument. CBCL, Child Behavior Checklist; SRS, Social Responsiveness Scale.

### Association between prenatal exposure to maternal SARS‐CoV‐2 infection and child neurodevelopment

Prenatal exposure to maternal SARS‐CoV‐2 infection was not associated with any neurodevelopmental outcome in the fully adjusted models (CBCL Internalizing: *β* = 0.04 [95% CI: −0.85, 0.88], *q* = 0.92; CBCL Externalizing: *β* = −0.31 [95% CI: −1.41, 0.87], *q* = 0.68; CBCL Total Problems: *β* = −0.50 [95% CI: −3.46, 2.51], *q* = 0.71; SRS: *β* = −0.60 [95% CI: −4.09, 3.17], *q* = 0.78) (Figure 4; Supporting Information S1: Appendices S10–S13).

**FIGURE 4:**
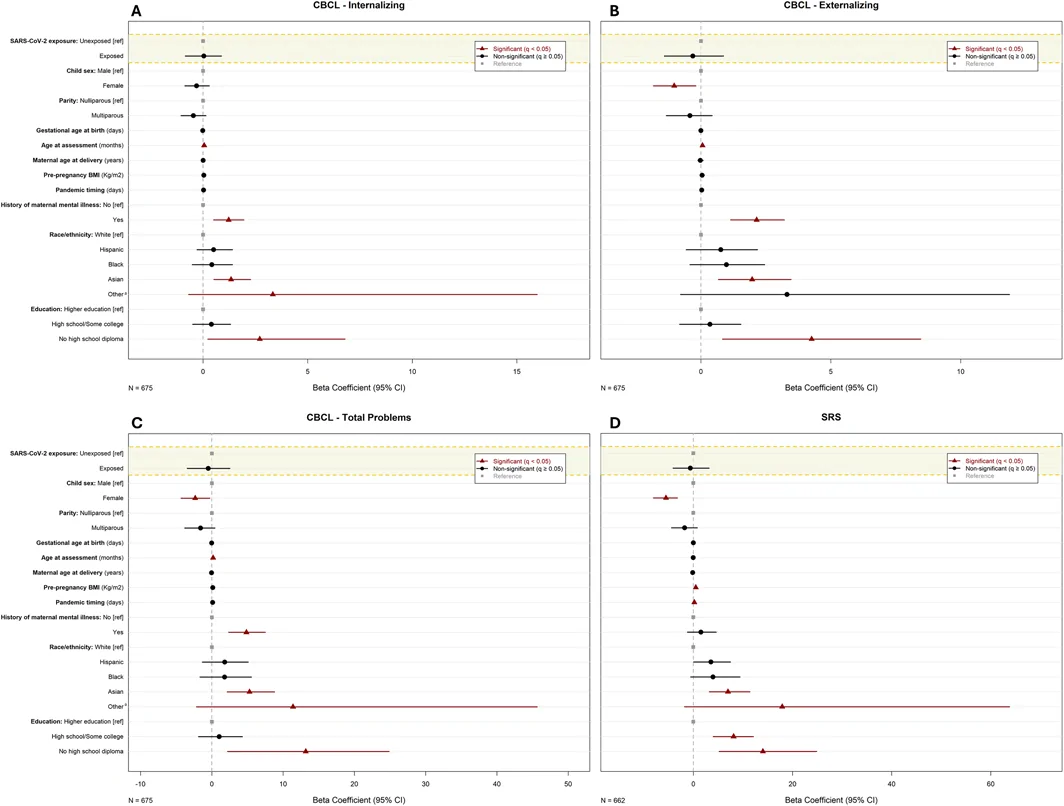
Association between prenatal exposure to SARS‐CoV‐2 infection and neurodevelopmental outcomes with covariates. Forest plots displaying Beta coefficients with 95% confidence intervals from multivariable robust regression models examining the associations between prenatal SARS‐CoV‐2 infection exposure and each neurodevelopmental outcome: CBCL—Internalizing (A), CBCL—Externalizing (B), CBCL—Total Problems (C), and SRS (D). The shaded area highlights the main exposure (SARS‐CoV‐2 infection); all the other covariates included in the analysis are listed below. Reference categories correspond to the largest groups. Positive values indicate higher scores. ^a^Due to the small sample size (*n* = 9) the Race/ethnicity: Other category yielded an unstable and asymmetrical estimate. BMI, body mass index; CBCL, Child Behavior Checklist; SRS, Social Responsiveness Scale.

Neither infection severity nor timing of infection was associated with any neurodevelopmental outcome. Results are reported in Supporting Information S1: Appendices S14 (Tables S2–S4; Figure S2) and S15 (Tables S5–S7; Figures S3–S6), respectively.

Sensitivity analyses excluding Gen C‐SF participants did not alter the results (Supporting Information S1: Appendix S16; Table S8; Figures S7, S9, and S10).

Stratifying by age at assessment (24–36, 37–48, and ≥49 months) showed no associations between prenatal exposure to SARS‐CoV‐2 infection and neurodevelopmental outcomes in any age group (Supporting Information S1: Appendix S17; Table S10).

### Association between prenatal exposure to maternal COVID‐19 vaccination and child neurodevelopment

Prenatal exposure to COVID‐19 vaccination was not associated with any neurodevelopmental outcome in the fully adjusted models (CBCL Internalizing: *β* = 0.41 [95% CI: −0.52, 1.44], *q* = 0.45; CBCL Externalizing: *β* = 1.42 [95% CI: −0.32, 2.95], *q* = 0.27; CBCL Total Problems: *β* = 2.13 [95% CI: −1.22, 6.15], *q* = 0.31; SRS: *β* = −3.65 [95% CI: −7.93, 0.32], *q* = 0.14) (Figure 1; Supporting Information S1: Appendices S18–S21).

Results were consistent in sensitivity analyses excluding Gen C‐SF participants (Supporting Information S1: Appendix S16; Table S9; Figure S8) and excluding participants vaccinated before pregnancy from the unvaccinated reference group (*n* = 52; Supporting Information S1: Appendices S22 and S23), with no associations in the fully adjusted models.

In the analyses stratified by age at assessment (24–48 and ≥49 months), prenatal exposure to vaccination was not associated with any outcome in either age group (Supporting Information S1: Appendix S17; Table S11).

### Covariate associations with neurodevelopmental outcomes

Adjustment for covariates revealed several significant associations with neurodevelopmental outcomes beyond the primary infection and vaccination exposures. Lower maternal educational attainment was associated with poorer outcomes across all four outcomes (β range: 2.71–14.03, *q* < 0.001). In all CBCL subscales, maternal history of mental illness (β range: 1.22–4.86, *q* < 0.001) and older child age at assessment (β range: 0.05–0.17, *q* < 0.01) were associated with poorer outcomes. Male child sex was associated with poorer outcomes on CBCL Externalizing and SRS (β range: −5.57 to −1.03, *q* < 0.001). Race/ethnicity was also associated with neurodevelopmental outcomes: identifying as Asian was associated with poorer outcomes in all CBCL subscales as well as SRS (β range: 1.30–7.14, *q* ≤ 0.001). Finally, poorer SRS outcomes were associated with higher maternal pre‐pregnancy BMI (β: 0.48, *q* < 0.001) and delivering later in the pandemic (β: 0.22, *q* < 0.01). Full covariate estimates from the associations between all neurodevelopmental outcomes and SARS‐CoV‐2 infection and COVID‐19 vaccination are reported in Supporting Information S1: Appendices S10–S13 and Supporting Information S1: Appendices S18–S21, respectively.

## DISCUSSION

In this sociodemographically diverse, prospective cohort of 675 children aged 2–6 years, we found no associations between prenatal exposure to SARS‐CoV‐2 infection or COVID‐19 vaccination and neurodevelopmental outcomes, including internalizing, externalizing, and total problem behaviors and social responsiveness, after robust covariate adjustment. While the COVID‐19 pandemic may no longer present an acute public health emergency, these findings support the absence of detectable adverse neurodevelopmental associations following prenatal SARS‐CoV‐2 infection or COVID‐19 vaccination.

These results are consistent with a growing body of literature reporting no associations between prenatal SARS‐CoV‐2 exposure and early childhood neurodevelopment (Firestein et al., 2023, 2024; Hardie, Marryat, et al., 2025; Jaswa, Huddleston, et al., 2024; Namakin et al., 2023; Pinheiro et al., 2024; Shuffrey et al., 2022; Vrantsidis et al., 2024), and, to our knowledge, extend this evidence to the longest follow‐up reported in the literature, assessing children up to 6 years of age. Age‐stratified analyses showed no evidence that associations differed by age at assessment, although estimates within age groups, particularly for children assessed at 49 months or older, were imprecise. Secondary analyses examining infection severity and gestational timing did not reveal increased risk of adverse neurodevelopment. However, these subgroup analyses were limited by small group sizes and should be interpreted with caution.

Importantly, exposed and unexposed groups differed substantially on characteristics known to influence neurodevelopmental trajectories. Mothers with SARS‐CoV‐2 infection during pregnancy were more likely to identify as Hispanic or Black, consistent with documented disparities in occupational exposure and healthcare access during the pandemic in New York City (Douglas & Subica, 2020; Janevic et al., 2022; Maroko et al., 2020), and to have higher pre‐pregnancy BMI and a history of mental illness, both established risk factors for adverse child neurodevelopment (Sanchez et al., 2018; Sindalovsky et al., 2025; Van Den Bergh et al., 2020). Infected mothers were more likely to be recruited from the Gen C‐SF cohort, reflecting later‐pandemic recruitment after widespread lifting of restrictions and potential exposure to differing viral variants. Maternal educational attainment and race/ethnicity were among the covariates most strongly associated with neurodevelopmental outcomes, with lower educational attainment and some minoritized racial/ethnic groups showing higher symptom scores across scales, consistent with prior research (Fraiman et al., 2022; Zhuo & Harrigan, 2023). Male sex and older age at assessment were also associated with worse outcomes independent of exposure, consistent with established developmental patterns (Breach & Lenz, 2023) and age‐related changes in symptom expression (Zheng et al., 2023). These findings have methodological implications beyond this cohort: the sociodemographic patterning of SARS‐CoV‐2 infection during pregnancy is non‐random, and insufficient adjustment for these factors risks attributing socially patterned developmental differences to prenatal infection. Our systematic review similarly identified inadequate covariate adjustment as a key source of bias in studies reporting adverse associations (Rizzo et al., 2025), and the comprehensive adjustment employed in the present study, including maternal health and socioeconomic factors often absent in prior work, reinforces that conclusion.

Prenatal exposure to maternal COVID‐19 vaccination was similarly not associated with adverse neurodevelopmental outcomes in this cohort, consistent with prior evidence (Favre et al., 2025; Hardie, Marryat, et al., 2025; Jaswa, Cedars, et al., 2024). The vaccinated group was small (*n* = 68), and vaccinated mothers were more often recruited through the later Gen C‐SF cohort (19.1% vs. 4.6%) and delivered later in the pandemic, reflecting evolving vaccine availability and acceptance during pregnancy. Vaccinated mothers also differed systematically from unvaccinated mothers, being older, more highly educated, and more likely to identify as White or Asian, which may limit generalizability and precision. Nonetheless, the results are consistent with the broader literature in finding no meaningful adverse neurodevelopmental associations of prenatal COVID‐19 vaccination.

Longitudinal follow‐up into school age will be essential, as some neurodevelopmental difficulties may only manifest as academic and cognitive demands increase. Moreover, parent‐reported measures may not capture subtle neurobiological differences that are not yet behaviorally expressed. Future work incorporating direct developmental assessments and objective neurophysiological measures, such as electroencephalography, may help clarify whether prenatal exposure is associated with latent neural differences in the absence of overt behavioral effects. More broadly, understanding how structural and psychosocial determinants of health influence early neurodevelopment remains a priority, particularly given their stronger associations with child outcomes compared with prenatal SARS‐CoV‐2 exposure in the present study.

The strengths of this study include its prospective cohort design, robust exposure ascertainment using multiple sources, a relatively long follow‐up period into early childhood, and comprehensive adjustment for key covariates frequently absent from prior studies, including maternal mental health and socioeconomic factors. The sociodemographically diverse sample ensures relevance to populations disproportionately affected by the pandemic. Limitations include reliance on parent‐reported outcomes rather than direct developmental assessment, the use of the CBCL 1½‐5 and SRS‐2 Preschool Form in some children outside their intended age ranges, limited power for subgroup analyses of infection timing and severity and for age‐stratified analyses, the use of a single assessment per child, which precluded examination of within‐child developmental change and will be addressed in longitudinal analyses as follow‐up continues, and the absence of a pre‐pandemic comparison group, which limits disentangling effects of infection from broader pandemic‐related exposures such as social restrictions and maternal stress (Edlow et al., 2023; Fajardo‐Martinez et al., 2024; Shuffrey et al., 2022). The single‐site hospital‐based urban sample may also limit generalizability to non‐urban populations.

## CONCLUSIONS

Prenatal exposure to maternal SARS‐CoV‐2 infection or COVID‐19 vaccination was not associated with adverse neurodevelopmental outcomes in preschool children followed up to 6 years of age. Sociodemographic and clinical factors, including maternal race/ethnicity, educational attainment, history of mental illness, pre‐pregnancy BMI, and child sex and age at assessment, showed stronger associations with neurodevelopmental outcomes than prenatal infection or vaccination exposure, underscoring the importance of rigorous confounder adjustment in this literature and highlighting persistent health inequities that are distinct from pandemic‐specific exposures. These findings offer some reassurance to individuals infected with SARS‐CoV‐2 during pregnancy and are consistent with the safety of COVID‐19 vaccination in pregnancy. Continued follow‐up into school age is warranted to determine whether these null associations persist as cognitive and academic demands increase.

## AUTHOR CONTRIBUTIONS

**Marco Rizzo**: Conceptualization; writing—original draft; methodology; software; formal analysis; investigation; data curation; visualization; writing—review and editing. **Rushna Tubassum**: Writing—review and editing; data curation; investigation. **Floriana Milazzo**: Writing—review and editing; data curation; investigation. **Yasemin Schmitt**: Writing—review and editing; data curation; investigation. **Whitney Lieb**: Writing—review and editing; supervision; project administration. **Maria De Las Mercedes Perez‐Rodriguez**: Funding acquisition; writing—review and editing; project administration; supervision. **Lot D. de Witte**: Writing—review and editing; conceptualization; funding acquisition. **Veerle Bergink**: Funding acquisition; writing—review and editing; conceptualization. **Anna‐Sophie Rommel**: Conceptualization; investigation; funding acquisition; writing—original draft; writing—review and editing; visualization; formal analysis; supervision.

## CONFLICT OF INTEREST STATEMENT

The authors declare no conflicts of interest.

## ETHICAL CONSIDERATIONS

The study was approved on 3rd September 2020 by the Institutional Review Board (IRB‐20‐00425) at the Icahn School of Medicine at Mount Sinai, reviewed by the US Centers for Disease Control and Prevention (CDC), and conducted in compliance with federal laws, CDC policies, and the Declaration of Helsinki. All participants in this study provided written informed consent for themselves and their children.

## Supporting information

Supporting Information S1

## Data Availability

The data that support the findings of this study are available on request from the corresponding author. The data are not publicly available due to privacy or ethical restrictions.
