# Supplementary material for: Prenatal SARS‐CoV‐2 infection, COVID‐19 vaccination, and preschool neurodevelopment: A confounder‐adjusted cohort study
Source: JCPP Adv. 2026 Nov 9:e70187. Online ahead of print. doi: 10.1002/jcv2.70187 (PMC13748764; doi:10.1002/jcv2.70187)
Supplement: Supplementary file 1 [file JCV2-9999-e70187-s001.docx]

**Contents**

[Appendix S1. Distribution of child date of birth by month and year	5](#_Toc244697227)

[Appendix S2. Correlation between first and second assessments of CBCL and SRS measurements	6](#_Toc244697228)

[Figure S1. Correlation between first and second assessments of CBCL and SRS measurements	7](#_Toc244697229)

[Table S1. Stability of CBCL and SRS scores between the first and second assessments	8](#_Toc244697230)

[Appendix S3. Directed acyclic graph for SARS-CoV-2 infection and COVID-19 vaccination exposures	9](#_Toc244697231)

[Appendix S4. Flowchart of exclusion criteria and eligible participants	10](#_Toc244697232)

[Appendix S5. Cohort sociodemographic and clinical characteristics and survey scores stratified by SARS-CoV-2 infection severity	11](#_Toc244697233)

[Appendix S6. Cohort sociodemographic and clinical characteristics and survey scores stratified by trimester of prenatal SARS-CoV-2 exposure	13](#_Toc244697234)

[Appendix S7. Prevalence of neurodevelopmental risk of delay for CBCL outcomes stratified by SARS-CoV-2 exposure	15](#_Toc244697235)

[Appendix S8. Prevalence of neurodevelopmental risk of delay for SRS outcomes stratified by SARS-CoV-2 exposure	16](#_Toc244697236)

[Appendix S9. Distribution of neurodevelopmental scores with skewness statistics	17](#_Toc244697237)

[Appendix S10. Linear regression results (HC3 robust standard errors) for the association between prenatal SARS-CoV-2 infection and CBCL Internalizing outcomes including all covariates	18](#_Toc244697238)

[Appendix S11. Linear regression results (HC3 robust standard errors) for the association between prenatal SARS-CoV-2 infection and CBCL Externalizing outcomes including all covariates	19](#_Toc244697239)

[Appendix S12. Linear regression results (HC3 robust standard errors) for the association between prenatal SARS-CoV-2 infection and CBCL Total Problems outcomes including all covariates	20](#_Toc244697240)

[Appendix S13. Linear regression results (HC3 robust standard errors) for the association between prenatal SARS-CoV-2 infection and SRS outcomes including all covariates	21](#_Toc244697241)

[Appendix S14. Secondary analysis: association between SARS-CoV-2 infection severity and neurodevelopmental outcomes	22](#_Toc244697242)

[14.1 Statistical analysis	22](#_Toc244697243)

[14.2 Results	22](#_Toc244697244)

[Table S2. Multivariable linear regression results (HC3 robust standard errors) for SARS-CoV-2 infection severity and CBCL and SRS outcomes	23](#_Toc244697245)

[Figure S2. Forest plot of the association between SARS-CoV-2 infection severity and neurodevelopmental outcomes	24](#_Toc244697246)

[Table S3. Omnibus F-tests for the effect of SARS-CoV-2 infection severity on neurodevelopmental outcomes	25](#_Toc244697247)

[Table S4. Linear trend tests in neurodevelopmental outcomes across increasing SARS-CoV-2 infection severity categories	26](#_Toc244697248)

[Appendix S15. Secondary analysis: association between gestational timing of SARS-CoV-2 infection exposure and neurodevelopmental outcomes	27](#_Toc244697249)

[15.1 Statistical analysis	27](#_Toc244697250)

[15.2 Results	27](#_Toc244697251)

[Table S5. Results from contrasts between exposure to SARS-CoV-2 infection during the third trimester (n=66) and no exposure (n=557) for each neurodevelopmental outcome	28](#_Toc244697252)

[Table S6. Results from contrasts between exposure to SARS-CoV-2 infection in the third trimester (n=66) vs first and second trimesters combined (n=52) for each neurodevelopmental outcome	29](#_Toc244697253)

[Table S7. Pairwise contrasts comparing all trimesters of SARS-CoV-2 infection exposure for each neurodevelopmental outcome	30](#_Toc244697254)

[Figure S3. Forest plots of the associations between gestational timing of SARS-CoV-2 infection and neurodevelopmental outcomes	31](#_Toc244697255)

[Figure S4. Stepwise attenuation of the SARS-CoV-2 infection exposure in the third vs second trimester with SRS outcome	32](#_Toc244697256)

[Figure S5. Maternal pre-pregnancy BMI distribution in the second and third trimesters of gestational SARS-CoV-2 infection	33](#_Toc244697257)

[Figure S6. Maternal race/ethnicity distribution and effects on SRS score difference between second and third trimesters of gestational SARS-CoV-2 infection	34](#_Toc244697258)

[Appendix S16. Sensitivity analysis excluding participants recruited from the Gen C-SF cohort	35](#_Toc244697259)

[16.1 SARS-CoV-2 infection	35](#_Toc244697260)

[**Table S8.** Linear regression results (HC3 robust standard errors) results of the association between prenatal exposure to SARS-CoV-2 infection and neurodevelopmental outcomes excluding participants recruited from the Gen C-SF cohort	35](#_Toc244697261)

[**Figure S7.** Sensitivity analysis: Forest plot of the association between prenatal exposure to SARS-CoV-2 infection and neurodevelopmental outcomes excluding participants recruited from the Gen C-SF cohort	36](#_Toc244697262)

[16.2 COVID-19 vaccination	37](#_Toc244697263)

[**Table S9.** Linear regression results (HC3 robust standard errors) results of the association between prenatal exposure to COVID-19 vaccination and neurodevelopmental outcomes excluding participants recruited from the Gen C-SF cohort	37](#_Toc244697264)

[**Figure S8.** Sensitivity analysis: Forest plot of the association between prenatal exposure to COVID-19 vaccination and neurodevelopmental outcomes excluding participants recruited from the Gen C-SF cohort	38](#_Toc244697265)

[16.3 Infection severity	39](#_Toc244697266)

[**Figure S9.** Sensitivity analysis: Forest plot of the association between SARS-CoV-2 infection severity and neurodevelopmental outcomes excluding participants recruited from the Gen C-SF cohort	39](#_Toc244697267)

[16.4 Infection timing	40](#_Toc244697268)

[**Figure S10.** Sensitivity analysis: Forest plots of the associations between gestational timing of SARS-CoV-2 infection and neurodevelopmental outcomes excluding participants recruited from the Gen C-SF cohort	40](#_Toc244697269)

[Appendix S17. Primary analysis stratified by age category	41](#_Toc244697270)

[17.1 Statistical analysis	41](#_Toc244697271)

[17.2 Results	41](#_Toc244697272)

[17.2.1 SARS-CoV-2 infection	41](#_Toc244697273)

[17.2.2 COVID-19 vaccination	41](#_Toc244697274)

[**Table S10.** Association between prenatal SARS-CoV-2 infection exposure and child neurodevelopmental outcomes stratified by age group at assessment	42](#_Toc244697275)

[**Table S11.** Association between prenatal COVID-19 vaccination exposure and child neurodevelopmental outcomes stratified by age group at assessment	43](#_Toc244697276)

[Appendix S18. Linear regression results (HC3 robust standard errors) for the association between prenatal COVID-19 vaccination and CBCL Internalizing outcomes including all covariates	44](#_Toc244697277)

[Appendix S19. Linear regression results (HC3 robust standard errors)for the association between prenatal COVID-19 vaccination and CBCL Externalizing outcomes including all covariates	45](#_Toc244697278)

[Appendix S20. Linear regression results (HC3 robust standard errors) for the association between prenatal COVID-19 vaccination and CBCL Total Problems outcomes including all covariates	46](#_Toc244697279)

[Appendix S21. Linear regression results (HC3 robust standard errors) for the association between prenatal COVID-19 vaccination and SRS outcomes including all covariates	47](#_Toc244697280)

[Appendix S22. Sensitivity analysis: results from the association between prenatal exposure to COVID-19 vaccination and neurodevelopmental outcomes excluding participants vaccinated before pregnancy	48](#_Toc244697281)

[Appendix S23. Sensitivity analysis: Forest plot of the association between prenatal exposure to COVID-19 vaccination and neurodevelopmental outcomes excluding participants vaccinated before pregnancy	49](#_Toc244697282)

# Appendix S1. Distribution of child date of birth by month and year

**Note:** Distribution of child date of birth by month and year across the study period for the full sample included in the study (N=675).

# Appendix S2. Correlation between first and second assessments of CBCL and SRS measurements

To evaluate whether the choice of assessment could influence our findings, we examined the agreement between the first (T1) and second (T2) assessments in children with two assessments (CBCL, n=37; SRS, n=29). For each outcome, we calculated the Pearson correlation, the intraclass correlation coefficient (ICC; absolute agreement), and the mean within-child change (T2 − T1) with 95% CIs.

Scores were strongly correlated across assessments (Pearson r = 0.76–0.86), with moderate to good agreement (ICC = 0.61–0.85). Mean CBCL scores were slightly higher at T2 (Internalizing +1.81 (0.16, 3.46); Externalizing +0.86 (-0.58, 2.31); Total Problems +3.84 (-0.12, 7.80)), whereas SRS scores were stable (+0.79 (-4.62, 6.20)) (Figure S1, Table S1).

The correlation results showed that children’s scores were largely preserved between assessments, so using the most recent assessment is unlikely to have reordered children within our sample. The modest increase in CBCL scores is consistent with developmental change over the interval between visits, which will be examined in longitudinal analyses as follow-up of this cohort continues.

## Figure S1. Correlation between first and second assessments of CBCL and SRS measurements

**Note:** Scatterplots of scores at the first (T1) versus second (T2) assessment among children with two assessments (CBCL, n=37; SRS, n=29). The dashed line indicates perfect agreement (T2 = T1) and the solid line the fitted linear trend.

r = Pearson correlation; ICC = intraclass correlation coefficient (absolute agreement); CBCL = Child Behavior Checklist; SRS = Social Responsiveness Scale

## Table S1. Stability of CBCL and SRS scores between the first and second assessments

| **Outcome** | **N** | **Pearson r** | **ICC** | **Mean change** |
| --- | --- | --- | --- | --- |
| **CBCL – Internalizing** | 37 | 0.77 (0.59, 0.88) | 0.61 (0.35, 0.78) | 1.81 (0.16, 3.46) |
| **CBCL – Externalizing** | 37 | 0.76 (0.58, 0.87) | 0.74 (0.55, 0.86) | 0.86 (-0.58, 2.31) |
| **CBCL – Total Problems** | 37 | 0.86 (0.73, 0.92) | 0.76 (0.58, 0.87) | 3.84 (-0.12, 7.80) |
| **SRS** | 29 | 0.85 (0.71, 0.93) | 0.85 (0.70, 0.93) | 0.79 (-4.62, 6.20) |

**Note:** Values are estimates (95% CI). ICC = intraclass correlation coefficient (absolute agreement). Mean change is the average within-child difference (T2 − T1).

CBCL = Child Behavior Checklist; SRS = Social Responsiveness Scale

# Appendix S3. Directed acyclic graph for SARS-CoV-2 infection and COVID-19 vaccination exposures

**Note:** Directed acyclic graphs (DAG) were constructed to guide covariate selection, minimize bias in the effect estimates, and inform causal inference for both SARS-CoV-2 infection (a) and COVID-19 vaccination (b) exposures. Covariates were classified based on their hypothesized roles as confounders or precision variables.

BMI = body mass index

# Appendix S4. Flowchart of exclusion criteria and eligible participants

CBCL = Child Behavior Checklist; SRS = Social Responsiveness Scale

# Appendix S5. Cohort sociodemographic and clinical characteristics and survey scores stratified by SARS-CoV-2 infection severity

| **Characteristic** | **Unexposed**  n = 557 | **Asymptomatic**  n = 60 | **Mild**  n = 37 | **Moderate/Severe**  n = 18 | **Unknown**  n = 3 | **p-value**^a^ |
| --- | --- | --- | --- | --- | --- | --- |
| **CBCL – Internalizing**  Median (Q1, Q3) | 2.0 (1.0, 6.0) | 3.0 (1.0, 8.0) | 3.0 (1.0, 6.0) | 0.5 (0.0, 9.0) | 2.0 (1.5, 2.0) | 0.33 |
| **CBCL – Externalizing**  Median (Q1, Q3) | 4.0 (1.0, 9.0) | 4.5 (2.0, 10.0) | 5.0 (1.0, 11.0) | 4.5 (0.2, 11.8) | 1.0 (1.0, 5.5) | 0.84 |
| **CBCL – Total Problems**  Median (Q1, Q3) | 11.0 (5.0, 23.0) | 14.5 (6.0, 28.2) | 14.0 (6.0, 23.0) | 8.5 (3.0, 35.2) | 3.0 (2.5, 8.0) | 0.60 |
| **SRS raw score^b^**  Median (Q1, Q3) | 28.0 (18.0, 40.0) | 33.0 (19.2, 49.0) | 25.0 (15.0, 44.0) | 25.0 (23.0, 37.0) | 34.0 (25.0, 42.5) | 0.48 |
| **Trimester of exposure**, n (%) |  |  |  |  |  | 0.06 |
| First trimester | NA | 7 (11.7%) | 5 (13.5%) | 4 (22.2%) | 1 (33.3%) |  |
| Second trimester |  | 12 (20.0%) | 16 (43.2%) | 7 (38.9%) | 0 (0.0%) |  |
| Third trimester |  | 41 (68.3%) | 16 (43.2%) | 7 (38.9%) | 2 (66.7%) |  |
| **Vaccine exposure**, n (%) |  |  |  |  |  | 0.23 |
| Exposed | 62 (11.1%) | 4 (6.7%) | 2 (5.4%) | 0 (0.0%) | 0 (0.0%) |  |
| Unexposed | 495 (88.9%) | 56 (93.3%) | 35 (94.6%) | 18 (100.0%) | 3 (100.0%) |  |
| **Maternal age at delivery** (years) |  |  |  |  |  | 0.45 |
| Median (Q1, Q3) | 34.0 (30.0, 37.0) | 32.0 (28.0, 36.0) | 33.0 (31.0, 36.0) | 32.0 (30.0, 38.8) | 30.0 (29.5, 33.5) |  |
| **Pandemic timing** (days) |  |  |  |  |  | **0.001** |
| Median (Q1, Q3) | 285.0 (155.0, 472.0) | 239.0 (142.0, 560.0) | 665.0 (228.0, 848.0) | 368.0 (183.0, 980.0) | 998.0 (973.5, 1003.5) |  |
| **Race/Ethnicity**, n (%) |  |  |  |  |  | **<0.001** |
| Asian | 74 (13.3%) | 2 (3.3%) | 2 (5.4%) | 1 (5.6%) | 0 (0.0%) |  |
| Black | 87 (15.6%) | 15 (25.0%) | 9 (24.3%) | 5 (27.8%) | 0 (0.0%) |  |
| Hispanic | 133 (23.9%) | 30 (50.0%) | 7 (18.9%) | 6 (33.3%) | 2 (66.7%) |  |
| White | 257 (46.1%) | 12 (20.0%) | 18 (48.6%) | 5 (27.8%) | 1 (33.3%) |  |
| Other | 6 (1.1%) | 1 (1.7%) | 1 (2.7%) | 1 (5.6%) | 0 (0.0%) |  |
| **Education**, n (%) |  |  |  |  |  | 0.07 |
| No high school diploma | 23 (4.1%) | 6 (10.0%) | 0 (0.0%) | 1 (5.6%) | 0 (0.0%) |  |
| High school/Some college | 144 (25.9%) | 22 (36.7%) | 11 (29.7%) | 3 (16.7%) | 1 (33.3%) |  |
| Higher education | 390 (70.0%) | 32 (53.3%) | 26 (70.3%) | 14 (77.8%) | 2 (66.7%) |  |
| **Maternal pre-pregnancy BMI** (kg/m^2^) |  |  |  |  |  | **0.001** |
| Median (Q1, Q3) | 25.2 (22.0, 30.2) | 29.3 (25.0, 33.6) | 26.9 (23.6, 32.8) | 28.0 (24.5, 29.7) | 33.9 (32.5, 36.0) |  |
| **Parity**, n (%) |  |  |  |  |  | **0.02** |
| Nulliparous | 294 (52.8%) | 24 (40.0%) | 16 (43.2%) | 4 (22.2%) | 0 (0.0%) |  |
| Multiparous | 263 (47.2%) | 36 (60.0%) | 21 (56.8%) | 14 (77.8%) | 3 (100.0%) |  |
| **History of maternal mental illness**, n (%) |  |  |  |  |  | **<0.001** |
| Yes | 203 (36.4%) | 28 (46.7%) | 27 (73.0%) | 3 (16.7%) | 1 (33.3%) |  |
| No | 354 (63.6%) | 32 (53.3%) | 10 (27.0%) | 15 (83.3%) | 2 (66.7%) |  |
| **Child sex**, n (%) |  |  |  |  |  | 0.23 |
| Female | 270 (48.5%) | 27 (45.0%) | 22 (59.5%) | 12 (66.7%) | 2 (66.7%) |  |
| Male | 287 (51.5%) | 33 (55.0%) | 15 (40.5%) | 6 (33.3%) | 1 (33.3%) |  |
| **Gestational age at birth** (days) |  |  |  |  |  | 0.87 |
| Median (Q1, Q3) | 274.0 (268.0, 279.0) | 274.0 (263.8, 279.2) | 274.0 (266.0, 276.0) | 274.5 (267.8, 277.0) | 276.0 (268.0, 288.0) |  |
| **Age at assessment** (years) |  |  |  |  |  | 0.11 |
| Median (Q1, Q3) | 3.2 (2.5, 3.8) | 3.3 (3.0, 3.7) | 3.4 (3.1, 3.7) | 3.7 (3.2, 3.9) | 3.4 (3.3, 3.6) |  |
| **Recruitment cohort**, n (%) |  |  |  |  |  | **<0.001** |
| Gen C | 533 (95.7%) | 58 (96.7%) | 32 (86.5%) | 11 (61.1%) | 0 (0.0%) |  |
| Gen C-SF | 24 (4.3%) | 2 (3.3%) | 5 (13.5%) | 7 (38.9%) | 3 (100.0%) |  |

^a^ Pearson's Chi-squared test; Kruskal-Wallis test. Significant p-values appear in bold.

^b^ For SRS outcomes, group sizes are Unexposed=548, Asymptomatic=58, Mild=36, Moderate/Severe=17, Unknown=3

BMI = body mass index; CBCL = Child Behavior Checklist; SRS = Social Responsiveness Scale; NA = not applicable

# Appendix S6. Cohort sociodemographic and clinical characteristics and survey scores stratified by trimester of prenatal SARS-CoV-2 exposure

| **Characteristic** | **Unexposed**  n = 557 | **First trimester**  n = 17 | **Second trimester**  n = 35 | **Third trimester**  n = 66 | **p-value**^a^ |
| --- | --- | --- | --- | --- | --- |
| **CBCL – Internalizing** |  |  |  |  | 0.19 |
| Median (Q1, Q3) | 2.0 (1.0, 6.0) | 3.0 (1.0, 8.0) | 2.0 (0.0, 4.0) | 3.0 (1.0, 8.0) |  |
| **CBCL – Externalizing** |  |  |  |  | 0.31 |
| Median (Q1, Q3) | 4.0 (1.0, 9.0) | 6.0 (2.0, 8.0) | 3.0 (1.0, 7.0) | 5.0 (2.0, 11.0) |  |
| **CBCL – Total Problems** |  |  |  |  | 0.47 |
| Median (Q1, Q3) | 11.0 (5.0, 23.0) | 14.0 (6.0, 22.0) | 10.0 (4.5, 18.0) | 15.0 (4.2, 29.8) |  |
| **SRS raw score^b^**  Median (Q1, Q3) | 28.0 (18.0, 40.0) | 29.0 (20.0, 45.0) | 21.0 (15.0, 28.0) | 38.0 (22.0, 51.0) | **<0.01** |
| **Disease severity**, n (%) |  |  |  |  | 0.08 |
| Asymptomatic | NA | 7 (41.2%) | 12 (34.3%) | 41 (62.1%) |  |
| Mild |  | 5 (29.4%) | 16 (45.7%) | 16 (24.2%) |  |
| Moderate/Severe |  | 4 (23.5%) | 7 (20.0%) | 7 (10.6%) |  |
| Unknown |  | 1 (5.9%) | 0 (0.0%) | 2 (3.0%) |  |
| **Vaccine exposure**, n (%) |  |  |  |  | 0.21 |
| Exposed | 62 (11.1%) | 0 (0.0%) | 2 (5.7%) | 4 (6.1%) |  |
| Unexposed | 495 (88.9%) | 17 (100.0%) | 33 (94.3%) | 62 (93.9%) |  |
| **Pandemic timing** (days)  Median (Q1, Q3) | 285.0 (155.0, 472.0) | 862.0 (273.0, 889.0) | 228.0 (175.0, 824.5) | 316.5 (133.2, 709.5) | **0.001** |
| **Maternal age at delivery** (years) |  |  |  |  | 0.56 |
| Median (Q1, Q3) | 34.0 (30.0, 37.0) | 32.0 (30.0, 36.0) | 33.0 (31.0, 36.0) | 32.0 (28.2, 36.0) |  |
| **Race/Ethnicity**, n (%) |  |  |  |  | **<0.001** |
| Asian | 74 (13.3%) | 1 (5.9%) | 2 (5.7%) | 2 (3.0%) |  |
| Black | 87 (15.6%) | 5 (29.4%) | 7 (20.0%) | 17 (25.8%) |  |
| Hispanic | 133 (23.9%) | 6 (35.3%) | 11 (31.4%) | 28 (42.4%) |  |
| White | 257 (46.1%) | 4 (23.5%) | 15 (42.9%) | 17 (25.8%) |  |
| Other | 6 (1.1%) | 1 (5.9%) | 0 (0.0%) | 2 (3.0%) |  |
| **Education**, n (%) |  |  |  |  | 0.08 |
| No high school diploma | 23 (4.1%) | 0 (0.0%) | 0 (0.0%) | 7 (10.6%) |  |
| High school/Some college | 144 (25.9%) | 7 (41.2%) | 10 (28.6%) | 20 (30.3%) |  |
| Higher education | 390 (70.0%) | 10 (58.8%) | 25 (71.4%) | 39 (59.1%) |  |
| **Maternal pre-pregnancy BMI** (kg/m^2^) |  |  |  |  | **<0.001** |
| Median (Q1, Q3) | 25.2 (22.0, 30.2) | 29.8 (26.0, 31.6) | 25.1 (21.8, 30.4) | 29.7 (25.8, 35.4) |  |
| **Parity**, n (%) |  |  |  |  | **0.02** |
| Nulliparous | 294 (52.8%) | 5 (29.4%) | 14 (40.0%) | 25 (37.9%) |  |
| Multiparous | 263 (47.2%) | 12 (70.6%) | 21 (60.0%) | 41 (62.1%) |  |
| **History of maternal mental illness**, n (%) |  |  |  |  | **0.03** |
| Yes | 203 (36.4%) | 10 (58.8%) | 15 (42.9%) | 34 (51.5%) |  |
| No | 354 (63.6%) | 7 (41.2%) | 20 (57.1%) | 32 (48.5%) |  |
| **Child sex**, n (%) |  |  |  |  | 0.48 |
| Female | 270 (48.5%) | 7 (41.2%) | 18 (51.4%) | 38 (57.6%) |  |
| Male | 287 (51.5%) | 10 (58.8%) | 17 (48.6%) | 28 (42.4%) |  |
| **Gestational age at birth** (days) |  |  |  |  | 0.50 |
| Median (Q1, Q3) | 274.0 (268.0, 279.0) | 276.0 (264.0, 283.0) | 274.0 (265.0, 277.0) | 274.0 (264.0, 277.8) |  |
| **Age at assessment** (years) |  |  |  |  | 0.09 |
| Median (Q1, Q3) | 3.2 (2.5, 3.8) | 3.2 (3.1, 3.4) | 3.4 (3.1, 3.6) | 3.4 (3.1, 3.8) |  |
| **Recruitment cohort**, n (%)  Gen C  Gen C-SF | 533 (95.7%)  24 (4.3%) | 14 (82.4%)  3 (17.6%) | 30 (85.7%)  5 (14.3%) | 57 (86.4%)  9 (13.6%) | **<0.001** |
| ^a^ Pearson's Chi-squared test; Kruskal-Wallis test. Significant p-values appear in bold.  ^b^ For SRS outcomes, group sizes are: Unexposed n=548, First trimester n=17, Second trimester n=33, Third trimester n=64.  BMI = body mass index; CBCL = Child Behavior Checklist; NA = not applicable; SRS = Social Responsiveness Scale | | | | | |
|  | | | | | |

# Appendix S7. Prevalence of neurodevelopmental risk of delay for CBCL outcomes stratified by SARS-CoV-2 exposure

|  | **Risk category** | | |
| --- | --- | --- | --- |
| **CBCL – Internalizing** | Typical | Borderline | Clinical |
| **Total** | 628 (93.0%) | 23 (3.4%) | 24 (3.6%) |
| **SARS-CoV-2 unexposed** | 522 (93.7%) | 16 (2.9%) | 19 (3.4%) |
| **SARS-CoV-2 exposed** | 106 (89.8%) | 7 (5.9%) | 5 (4.2%) |
|  |  | **Risk category** |  |
| **CBCL – Externalizing** | Typical | Borderline | Clinical |
| **Total** | 642 (95.1%) | 17 (2.5%) | 16 (2.4%) |
| **SARS-CoV-2 unexposed** | 530 (95.2%) | 15 (2.7%) | 12 (2.2%) |
| **SARS-CoV-2 exposed** | 112 (94.9%) | 2 (1.7%) | 4 (3.4%) |
|  |  | **Risk category** |  |
| **CBCL – Total Problems** | Typical | Borderline | Clinical |
| **Total** | 638 (94.5%) | 13 (1.9%) | 24 (3.6%) |
| **SARS-CoV-2 unexposed** | 529 (95.0%) | 10 (1.8%) | 18 (3.2%) |
| **SARS-CoV-2 exposed** | 109 (92.4%) | 3 (2.5%) | 6 (5.1%) |

**Note:** Risk categories follow clinical cutoffs for the CBCL Internalizing, Externalizing, and Total Problems composite subscales. Data are reported as n (%).

CBCL = Child Behavior Checklist

# Appendix S8. Prevalence of neurodevelopmental risk of delay for SRS outcomes stratified by SARS-CoV-2 exposure

|  | **Risk category** | | | |
| --- | --- | --- | --- | --- |
| **SRS** | Typical | Mild | Moderate | Severe |
| **Total** | 618 (93.4%) | 23 (3.5%) | 12 (1.8%) | 9 (1.4%) |
| **SARS-CoV-2 unexposed** | 516 (94.2%) | 15 (2.7%) | 10 (1.8%) | 7 (1.3%) |
| **SARS-CoV-2 exposed** | 102 (89.5%) | 8 (7.0%) | 2 (1.8%) | 2 (1.8%) |

**Note:** Risk categories follow clinical cutoffs for the SRS. Data are reported as n (%).

SRS = Social Responsiveness Scale

# Appendix S9. Distribution of neurodevelopmental scores with skewness statistics

**Note:** Histograms with overlaid density curves (solid red) and mean lines (dotted blue). Each panel displays the skewness value for the respective distribution, and all histograms are plotted with frequency density. Skewness values indicate moderate to substantial deviation from normal distribution, supporting the use of linear regression (HC3 robust standard errors) methods for all analyses.

CBCL = Child Behavior Checklist; SRS = Social Responsiveness Scale

# Appendix S10. Linear regression results (HC3 robust standard errors) for the association between prenatal SARS-CoV-2 infection and CBCL Internalizing outcomes including all covariates

|  |  | **Minimally adjusted** | | | | **Fully adjusted** | | | |
| --- | --- | --- | --- | --- | --- | --- | --- | --- | --- |
| **Variable** | **n** | Beta | 95% CI | p-value | q-value | Beta | 95% CI | p-value | q-value |
| **SARS-CoV-2 exposure:** Unexposed [ref] | 557 |  |  |  |  |  |  |  |  |
| Exposed | 118 | 0.19 | (-0.66, 1.05) | 0.62 | 0.62 | 0.04 | (-0.85, 0.88) | 0.92 | 0.92 |
| **Child sex:** Male [ref] | 342 |  |  |  |  |  |  |  |  |
| Female | 333 | -0.28 | (-0.84, 0.30) | 0.32 | 0.38 | -0.31 | (-0.87, 0.30) | 0.27 | 0.37 |
| **Parity:** Nulliparous [ref] | 338 |  |  |  |  |  |  |  |  |
| Multiparous | 337 | -0.55 | (-1.15, 0.04) | 0.07 | 0.13 | -0.46 | (-1.05, 0.14) | 0.13 | 0.28 |
| **Gestational age at birth** (days) | 675 | - | - | - | - | -0.01 | (-0.04, 0.02) | 0.27 | 0.37 |
| **Age at assessment** (months) | 675 | 0.04 | (0.01, 0.07) | <0.01 | **0.02** | 0.05 | (0.02, 0.08) | <0.001 | **<0.01** |
| **Maternal age at delivery** (years) | 675 | 0.02 | (-0.05, 0.09) | 0.59 | 0.62 | 0.01 | (-0.06, 0.07) | 0.92 | 0.92 |
| **Pre-pregnancy BMI** (kg/m^2^) | 675 | - | - | - | - | 0.04 | (-0.03, 0.10) | 0.12 | 0.28 |
| **Pandemic timing** (days) | 675 | 0.04 | (0.01, 0.08) | <0.01 | **0.02** | 0.02 | (-0.02, 0.06) | 0.23 | 0.37 |
| **History of maternal mental illness:** No [ref] | 413 |  |  |  |  |  |  |  |  |
| Yes | 262 | - | - | - | - | 1.22 | (0.50, 1.95) | <0.001 | **<0.01** |
| **Race/ethnicity:** White [ref] | 293 |  |  |  |  |  |  |  |  |
| Hispanic | 178 | 0.73 | (-0.02, 1.52) | 0.08 | 0.13 | 0.50 | (-0.29, 1.40) | 0.23 | 0.37 |
| Black | 116 | 0.60 | (-0.32, 1.57) | 0.19 | 0.25 | 0.41 | (-0.52, 1.40) | 0.38 | 0.43 |
| Asian | 79 | 1.20 | (0.39, 2.20) | 0.01 | **0.02** | 1.34 | (0.52, 2.27) | <0.01 | **0.02** |
| Other^a^ | 9 | 3.35 | (-0.51, 15.80) | <0.01 | 0.02 | 3.33 | (-0.69, 15.97) | <0.01 | 0.02 |
| **Educational attainment:** Higher education [ref] | 464 |  |  |  |  |  |  |  |  |
| High school/Some college | 181 | 0.57 | (-0.32, 1.52) | 0.16 | 0.24 | 0.39 | (-0.50, 1.31) | 0.33 | 0.42 |
| No high school diploma | 30 | 2.93 | (0.51, 7.12) | <0.001 | **0.001** | 2.71 | (0.22, 6.79) | <0.001 | **<0.01** |

**Note:** Minimally adjusted models included: child sex, age at assessment, maternal education, race/ethnicity, maternal age at delivery, pandemic timing, and parity. Fully adjusted models additionally included pre-pregnancy BMI, maternal mental health history, and gestational age at birth. For each covariate, the largest group served as the reference with Beta set to 0. Statistical significance (FDR-adjusted q<0.05) is indicated in bold.

^a^Due to the small sample size (n=9), the Race/ethnicity: Other category yielded an unstable and asymmetrical estimate, and this category was therefore treated as non-significant.

BMI = body mass index

# Appendix S11. Linear regression results (HC3 robust standard errors) for the association between prenatal SARS-CoV-2 infection and CBCL Externalizing outcomes including all covariates

|  |  | **Minimally adjusted** | | | | **Fully adjusted** | | | |
| --- | --- | --- | --- | --- | --- | --- | --- | --- | --- |
| **Variable** | **n** | Beta | 95% CI | p-value | q-value | Beta | 95% CI | p-value | q-value |
| **SARS-CoV-2 exposure:** Unexposed [ref] | 557 |  |  |  |  |  |  |  |  |
| Exposed | 118 | -0.08 | (-1.16, 1.11) | 0.89 | 0.94 | -0.31 | (-1.41, 0.87) | 0.59 | 0.68 |
| **Child sex:** Male [ref] | 342 |  |  |  |  |  |  |  |  |
| Female | 333 | -0.95 | (-1.72, -0.13) | 0.03 | 0.06 | -1.03 | (-1.83, -0.20) | 0.01 | **0.04** |
| **Parity:** Nulliparous [ref] | 338 |  |  |  |  |  |  |  |  |
| Multiparous | 337 | -0.55 | (-1.46, 0.34) | 0.23 | 0.30 | -0.42 | (-1.33, 0.43) | 0.34 | 0.47 |
| **Gestational age at birth** (days) | 675 | - | - | - | - | 0.00 | (-0.05, 0.04) | 0.96 | 0.96 |
| **Age at assessment** (months) | 675 | 0.05 | (0.00, 0.09) | 0.03 | 0.06 | 0.06 | (0.01, 0.11) | <0.01 | **0.02** |
| **Maternal age at delivery** (years) | 675 | 0.00 | (-0.09, 0.10) | 0.94 | 0.94 | -0.02 | (-0.12, 0.08) | 0.66 | 0.71 |
| **Pre-pregnancy BMI** (kg/m^2^) | 675 | - | - | - | - | 0.05 | (-0.03, 0.13) | 0.20 | 0.36 |
| **Pandemic timing** (days) | 675 | 0.07 | (0.02, 0.12) | <0.01 | **0.03** | 0.03 | (-0.02, 0.08) | 0.25 | 0.38 |
| **History of maternal mental illness:** No [ref] | 413 |  |  |  |  |  |  |  |  |
| Yes | 262 | - | - | - | - | 2.15 | (1.14, 3.21) | <0.001 | **<0.001** |
| **Race/ethnicity:** White [ref] | 293 |  |  |  |  |  |  |  |  |
| Hispanic | 178 | 0.88 | (-0.24, 2.11) | 0.15 | 0.23 | 0.77 | (-0.57, 2.18) | 0.22 | 0.36 |
| Black | 116 | 1.09 | (-0.16, 2.49) | 0.11 | 0.21 | 0.98 | (-0.42, 2.45) | 0.16 | 0.34 |
| Asian | 79 | 1.67 | (0.31, 3.23) | 0.02 | 0.06 | 1.97 | (0.68, 3.47) | <0.01 | **0.02** |
| Other^a^ | 9 | 2.89 | (-1.07, 12.44) | 0.12 | 0.21 | 3.31 | (-0.79, 11.86) | 0.07 | 0.18 |
| **Educational attainment:** Higher education [ref] | 464 |  |  |  |  |  |  |  |  |
| High school/Some college | 181 | 0.62 | (-0.57, 1.89) | 0.31 | 0.37 | 0.35 | (-0.82, 1.54) | 0.57 | 0.68 |
| No high school diploma | 30 | 4.52 | (1.08, 9.04) | <0.001 | **<0.001** | 4.26 | (0.83, 8.46) | <0.001 | **<0.001** |

**Note:** Minimally adjusted models included: child sex, age at assessment, maternal education, race and ethnicity, maternal age at delivery, pandemic timing, and parity. Fully adjusted models additionally included pre-pregnancy BMI, maternal mental health history, and gestational age at birth. For each covariate, the largest group served as the reference with Beta set to 0. Statistical significance (FDR-adjusted q<0.05) is indicated in bold.

^a^Due to the small sample size (n=9), the Race/ethnicity: Other category yielded an unstable and asymmetrical estimate, and this category was therefore treated as non-significant.

BMI = body mass index

# Appendix S12. Linear regression results (HC3 robust standard errors) for the association between prenatal SARS-CoV-2 infection and CBCL Total Problems outcomes including all covariates

|  |  | **Minimally adjusted** | | | | **Fully adjusted** | | | |
| --- | --- | --- | --- | --- | --- | --- | --- | --- | --- |
| **Variable** | **n** | Beta | 95% CI | p-value | q-value | Beta | 95% CI | p-value | q-value |
| **SARS-CoV-2 exposure:** Unexposed [ref] | 557 |  |  |  |  |  |  |  |  |
| Exposed | 118 | 0.11 | (-2.70, 3.13) | 0.94 | 0.95 | -0.50 | (-3.46, 2.51) | 0.71 | 0.71 |
| **Child sex:** Male [ref] | 342 |  |  |  |  |  |  |  |  |
| Female | 333 | -2.16 | (-4.13, -0.09) | 0.03 | 0.07 | -2.34 | (-4.32, -0.29) | 0.02 | 0.05 |
| **Parity:** Nulliparous [ref] | 338 |  |  |  |  |  |  |  |  |
| Multiparous | 337 | -1.93 | (-4.05, 0.03) | 0.08 | 0.13 | -1.59 | (-3.80, 0.42) | 0.14 | 0.26 |
| **Gestational age at birth** (days) | 675 | - | - | - | - | -0.03 | (-0.14, 0.09) | 0.44 | 0.55 |
| **Age at assessment** (months) | 675 | 0.14 | (0.04, 0.25) | <0.01 | **0.02** | 0.17 | (0.06, 0.27) | <0.01 | **<0.01** |
| **Maternal age at delivery** (years) | 675 | -0.01 | (-0.24, 0.24) | 0.95 | 0.95 | -0.05 | (-0.29, 0.19) | 0.69 | 0.71 |
| **Pre-pregnancy BMI** (kg/m^2^) | 675 | - | - | - | - | 0.12 | (-0.09, 0.37) | 0.16 | 0.26 |
| **Pandemic timing** (days) | 675 | 0.20 | (0.08, 0.32) | <0.001 | **<0.01** | 0.11 | (-0.02, 0.24) | 0.08 | 0.16 |
| **History of maternal mental illness:** No [ref] | 413 |  |  |  |  |  |  |  |  |
| Yes | 262 | - | - | - | - | 4.84 | (2.37, 7.51) | <0.001 | **<0.001** |
| **Race/ethnicity:** White [ref] | 293 |  |  |  |  |  |  |  |  |
| Hispanic | 178 | 2.39 | (-0.55, 5.37) | 0.10 | 0.15 | 1.80 | (-1.33, 5.09) | 0.22 | 0.34 |
| Black | 116 | 2.35 | (-1.03, 5.92) | 0.15 | 0.20 | 1.78 | (-1.67, 5.53) | 0.29 | 0.39 |
| Asian | 79 | 4.64 | (1.36, 8.36) | <0.01 | **0.02** | 5.28 | (2.14, 8.79) | 0.001 | **<0.01** |
| Other^a^ | 9 | 10.79 | (-2.42, 45.76) | 0.02 | 0.04 | 11.38 | (-2.17, 45.63) | 0.01 | 0.03 |
| **Educational attainment:** Higher education [ref] | 464 |  |  |  |  |  |  |  |  |
| High school/Some college | 181 | 1.60 | (-1.44, 4.93) | 0.28 | 0.33 | 1.04 | (-1.87, 4.27) | 0.47 | 0.55 |
| No high school diploma | 30 | 13.78 | (2.84, 26.42) | <0.001 | **<0.001** | 13.17 | (2.19, 24.87) | <0.001 | **<0.001** |

**Note:** Minimally adjusted models included: child sex, age at assessment, maternal education, race and ethnicity, maternal age at delivery, pandemic timing, and parity. Fully adjusted models additionally included pre-pregnancy BMI, maternal mental health history, and gestational age at birth. For each covariate, the largest group served as the reference with Beta set to 0. Statistical significance (FDR-adjusted q<0.05) is indicated in bold.

^a^Due to the small sample size (n=9), the Race/ethnicity: Other category yielded an unstable and asymmetrical estimate, and this category was therefore treated as non-significant.

BMI = body mass index

# Appendix S13. Linear regression results (HC3 robust standard errors) for the association between prenatal SARS-CoV-2 infection and SRS outcomes including all covariates

|  |  | **Minimally adjusted** | | | | **Fully adjusted** | | | |
| --- | --- | --- | --- | --- | --- | --- | --- | --- | --- |
| **Variable** | **n** | Beta | 95% CI | p-value | q-value | Beta | 95% CI | p-value | q-value |
| **SARS-CoV-2 exposure:** Unexposed [ref] | 548 |  |  |  |  |  |  |  |  |
| Exposed | 114 | 0.14 | (-3.47, 4.01) | 0.93 | 0.93 | -0.60 | (-4.09, 3.17) | 0.72 | 0.78 |
| **Child sex:** Male [ref] | 338 |  |  |  |  |  |  |  |  |
| Female | 324 | -5.22 | (-7.78, -2.85) | <0.001 | **<0.001** | -5.52 | (-8.04, -3.25) | <0.001 | **<0.001** |
| **Parity:** Nulliparous [ref] | 331 |  |  |  |  |  |  |  |  |
| Multiparous | 331 | -1.54 | (-4.27, 0.95) | 0.24 | 0.32 | -1.76 | (-4.40, 0.78) | 0.18 | 0.26 |
| **Gestational age at birth** (days) | 662 | - | - | - | - | 0.01 | (-0.12, 0.12) | 0.81 | 0.82 |
| **Age at assessment** (months) | 662 | -0.04 | (-0.18, 0.09) | 0.47 | 0.56 | -0.04 | (-0.17, 0.10) | 0.59 | 0.68 |
| **Maternal age at delivery** (years) | 662 | -0.08 | (-0.36, 0.22) | 0.57 | 0.62 | -0.14 | (-0.43, 0.16) | 0.33 | 0.41 |
| **Pre-pregnancy BMI** (kg/m^2^) | 662 | - | - | - | - | 0.48 | (0.25, 0.73) | <0.001 | **<0.001** |
| **Pandemic timing** (days) | 662 | 0.22 | (0.06, 0.36) | <0.01 | **<0.01** | 0.19 | (0.02, 0.35) | 0.01 | **0.02** |
| **History of maternal mental illness:** No [ref] | 403 |  |  |  |  |  |  |  |  |
| Yes | 259 | - | - | - | - | 1.53 | (-1.18, 4.59) | 0.26 | 0.35 |
| **Race/ethnicity:** White [ref] | 287 |  |  |  |  |  |  |  |  |
| Hispanic | 173 | 5.82 | (2.58, 9.39) | 0.001 | **<0.01** | 3.53 | (0.07, 7.49) | 0.05 | 0.08 |
| Black | 115 | 7.07 | (3.16, 11.86) | <0.001 | **0.001** | 3.98 | (-0.55, 9.41) | 0.05 | 0.08 |
| Asian | 78 | 6.68 | (2.82, 10.90) | 0.001 | **<0.01** | 6.98 | (3.23, 11.40) | <0.001 | **<0.01** |
| Other^a^ | 9 | 18.57 | (0.53, 73.49) | <0.001 | <0.01 | 17.92 | (-1.79, 63.76) | <0.001 | <0.01 |
| **Educational attainment:** Higher education [ref] | 458 |  |  |  |  |  |  |  |  |
| High school/Some college | 175 | 9.21 | (5.13, 13.27) | <0.001 | **<0.001** | 8.12 | (4.03, 12.11) | <0.001 | **<0.001** |
| No high school diploma | 29 | 15.15 | (6.27, 25.90) | <0.001 | **<0.001** | 14.03 | (5.21, 24.88) | <0.001 | **<0.001** |

**Note:** Minimally adjusted models included: child sex, age at assessment, maternal education, race and ethnicity, maternal age at delivery, pandemic timing, and parity. Fully adjusted models additionally included pre-pregnancy BMI, maternal mental health history, and gestational age at birth. For each covariate, the largest group served as the reference with Beta set to 0. Statistical significance (FDR-adjusted q<0.05) is indicated in bold.

^a^Due to the small sample size (n=9), the Race/ethnicity: Other category yielded an unstable and asymmetrical estimate, and this category was therefore treated as non-significant.

BMI = body mass index

# Appendix S14. Secondary analysis: association between SARS-CoV-2 infection severity and neurodevelopmental outcomes

## 14.1 Statistical analysis

To evaluate whether neurodevelopmental outcomes vary by clinical severity of maternal SARS-CoV-2 infection, we fitted multivariable linear regression models with robust (HC3) standard errors for each outcome (CBCL Internalizing, Externalizing, Total Problems, and Social Responsiveness Scale scores), with exposure categorized into four levels: i) unexposed, ii) asymptomatic, iii) mild, and iv) moderate/severe. Severity was entered as a categorical variable with the unexposed group as the reference category, yielding severity-specific estimates versus no infection. Additionally, we reported an omnibus test to assess whether outcomes differ across severity levels, and a trend test to evaluate evidence of severity gradient.

## 14.2 Results

Analyses stratified by infection severity yielded no significant associations with any neurodevelopmental outcome across category-specific comparisons (Table S2; Figure S2), omnibus tests (Table S3) and severity gradient analysis (Table S4) for either minimally or fully adjusted models.

## Table S2. Multivariable linear regression results (HC3 robust standard errors) for SARS-CoV-2 infection severity and CBCL and SRS outcomes

|  |  |  | **Minimally adjusted** | | | **Fully adjusted** | | |
| --- | --- | --- | --- | --- | --- | --- | --- | --- |
| **Outcome** | **Infection severity groups** | **n** | Beta (95% CI) | p-value | q-value | Beta (95% CI) | p-value | q-value |
| **CBCL – Internalizing** | **Asymptomatic** | 60 | 0.41 (-0.60, 1.41) | 0.43 | 0.97 | 0.22 (-0.77, 1.22) | 0.66 | 0.97 |
|  | **Mild** | 37 | 0.34 (-0.91, 1.59) | 0.59 | 0.97 | 0.03 (-1.22, 1.28) | 0.96 | 0.97 |
|  | **Moderate/Severe** | 18 | -0.55 (-2.32, 1.21) | 0.54 | 0.97 | -0.24 (-2.00, 1.51) | 0.79 | 0.97 |
| **CBCL – Externalizing** | **Asymptomatic** | 60 | -0.29 (-1.80, 1.22) | 0.71 | 0.97 | -0.56 (-2.03, 0.92) | 0.46 | 0.97 |
|  | **Mild** | 37 | 0.20 (-1.67, 2.07) | 0.83 | 0.97 | -0.34 (-2.19, 1.50) | 0.72 | 0.97 |
|  | **Moderate/Severe** | 18 | 0.32 (-2.33, 2.96) | 0.81 | 0.97 | 0.89 (-1.70, 3.49) | 0.50 | 0.97 |
| **CBCL – Total Problems** | **Asymptomatic** | 60 | 0.82 (-2.79, 4.42) | 0.66 | 0.97 | 0.16 (-3.98, 3.72) | 0.93 | 0.97 |
|  | **Mild** | 37 | 0.29 (-4.19, 4.78) | 0.90 | 0.97 | -1.08 (-5.53, 3.38) | 0.64 | 0.97 |
|  | **Moderate/Severe** | 18 | -1.15 (-7.48, 5.20) | 0.72 | 0.97 | 0.13 (-6.14, 6.39) | 0.97 | 0.97 |
| **SRS^a^** | **Asymptomatic** | 58 | 0.85 (-3.53, 5.23) | 0.70 | 0.97 | 0.21 (-4.14, 4.57) | 0.92 | 0.97 |
|  | **Mild** | 36 | -0.77 (-6.19, 4.66) | 0.77 | 0.97 | -1.86 (-7.28, 3.57) | 0.50 | 0.97 |
|  | **Moderate/Severe** | 17 | -0.63 (-8.41, 7.16) | 0.78 | 0.97 | -0.50 (-8.28, 7.28) | 0.90 | 0.97 |

**Note:** Minimally adjusted models included: child sex, age at assessment, maternal education, race and ethnicity, maternal age at delivery, pandemic timing, and parity. Fully adjusted models additionally included pre-pregnancy BMI, maternal mental health history, and gestational age at birth. The reference group for all severity comparisons was the unexposed group (n = 557; no prenatal SARS-CoV-2 exposure).

^a^Unexposed n for SRS outcomes = 548

BMI = body mass index; CBCL = Child Behavior Checklist; SRS = Social Responsiveness Scale

## Figure S2. Forest plot of the association between SARS-CoV-2 infection severity and neurodevelopmental outcomes

**Note:** Forest plot displaying beta coefficients (95% confidence intervals) from multivariable linear regression (HC3 robust standard errors) models comparing each SARS-CoV-2 infection severity group (i.e., Asymptomatic in blue, Mild in dark red, Moderate/Severe in yellow) to the unexposed reference group (vertical dashed grey line at β=0). Only results from fully adjusted models are reported.

CBCL = Child Behavior Checklist; SRS = Social Responsiveness Scale

## Table S3. Omnibus F-tests for the effect of SARS-CoV-2 infection severity on neurodevelopmental outcomes

|  |  | **Minimally adjusted** | | | **Fully adjusted** | | |
| --- | --- | --- | --- | --- | --- | --- | --- |
| **Outcome** | **N** | F (df1, df2) | p-value | q-value | F (df1, df2) | p-value | q-value |
| **CBCL – Internalizing** | 672 | 0.46 (3, 657) | 0.71 | .97 | 0.10 (3, 654) | 0.96 | 0.97 |
| **CBCL – Externalizing** | 672 | 0.09 (3, 657) | 0.97 | .97 | 0.40 (3, 654) | 0.75 | 0.97 |
| **CBCL – Total Problems** | 672 | 0.12 (3, 657) | 0.95 | .97 | 0.08 (3, 654) | 0.97 | 0.97 |
| **SRS** | 659 | 0.09 (3, 644) | 0.97 | .97 | 0.16 (3, 641) | 0.92 | 0.97 |
| **Note:** Values are F-statistics (degrees of freedom) from multivariable linear regression (HC3 robust standard errors) models examining whether infection severity (Asymptomatic, Mild, Moderate/Severe) jointly predicts neurodevelopmental outcomes. Minimally adjusted models included: child sex, age at assessment, maternal education, race and ethnicity, maternal age at delivery, pandemic timing, and parity. Fully adjusted models additionally included pre-pregnancy BMI, maternal mental health history, and gestational age at birth. Results do not include participants with unknown information on severity of infection (n = 3).  BMI = body mass index; CBCL = Child Behavior Checklist; SRS = Social Responsiveness Scale | | | | | | | |

## Table S4. Linear trend tests in neurodevelopmental outcomes across increasing SARS-CoV-2 infection severity categories

|  |  | |  | | **All groups** | | | | | | | | |
| --- | --- | --- | --- | --- | --- | --- | --- | --- | --- | --- | --- | --- | --- |
|  | |  | | **Minimally adjusted** | | | | | **Fully adjusted** | | | | |
| **Outcome** | | **n** | | Beta | | 95% CI | p-value | q-value | Beta | 95% CI | p-value | | q-value |
| **CBCL – Internalizing** | | 672 | | 0.20 | | (-0.49, 0.89) | 0.57 | 0.94 | 0.13 | (-0.54, 0.81) | 0.70 | | 0.94 |
| **CBCL – Externalizing** | | 672 | | 0.13 | | (-0.65, 0.92) | 0.74 | 0.94 | 0.06 | (-0.71, 0.84) | 0.87 | | 0.94 |
| **CBCL – Total Problems** | | 672 | | 0.54 | | (-1.61, 2.68) | 0.62 | 0.94 | 0.31 | (-1.80, 2.42) | 0.77 | | 0.94 |
| **SRS** | | 659 | | 0.45 | | (-1.77, 2.67) | 0.69 | 0.94 | 0.16 | (-2.02, 2.34) | 0.88 | | 0.94 |
|  |  | |  | |  | | | | | | |  | |
|  |  | |  | | **SARS-CoV-2 exposed only** | | | | | | | | |
|  | |  | | **Minimally adjusted** | | | | | **Fully adjusted** | | | | |
| **Outcome** | | **n** | | Beta | | 95% CI | p-value | q-value | Beta | 95% CI | p-value | | q-value |
| **CBCL – Internalizing** | | 115 | | 0.00 | | (-1.86, 1.86) | 0.99 | 0.99 | 0.42 | (-1.46, 2.30) | 0.66 | | 0.94 |
| **CBCL – Externalizing** | | 115 | | 0.70 | | (-1.35, 2.74) | 0.50 | 0.94 | 1.27 | (-0.77, 3.31) | 0.22 | | 0.94 |
| **CBCL – Total Problems** | | 115 | | 1.19 | | (-4.55, 6.93) | 0.68 | 0.94 | 2.74 | (-3.02, 8.50) | 0.35 | | 0.94 |
| **SRS** | | 111 | | 0.95 | | (-4.65, 6.55) | 0.74 | 0.94 | 1.76 | (-3.92, 7.43) | 0.54 | | 0.94 |

**Note:** Groups sizes: SARS-CoV-2 unexposed n=557, Asymptomatic n=60, Mild n=37, Moderate/Severe n=18 for CBCL and SARS-CoV-2 unexposed n=548, Asymptomatic n=58, Mild n=36, Moderate/Severe n=17 for SRS. Results do not include participants with unknown information on severity of infection (n = 3). "All groups" included the SARS-CoV-2 unexposed group as the reference. "SARS-CoV-2 exposed only" examined the gradient among exposed participants. Beta coefficients represent the change in outcome per one-unit increase in severity. Minimally adjusted models included: child sex, age at assessment, maternal education, race and ethnicity, maternal age at delivery, pandemic timing, and parity. Fully adjusted models additionally included pre-pregnancy BMI, maternal mental health history, and gestational age at birth.

BMI = body mass index; CBCL = Child Behavior Checklist; SRS = Social Responsiveness Scale

# Appendix S15. Secondary analysis: association between gestational timing of SARS-CoV-2 infection exposure and neurodevelopmental outcomes

## 15.1 Statistical analysis

To evaluate whether gestational timing of maternal SARS-CoV-2 infection was associated with child neurodevelopmental outcomes, multivariable linear regression models with robust (HC3) standard errors were fitted for each outcome (CBCL Internalizing, Externalizing, Total Problems, and Social Responsiveness Scale scores). Gestational timing was operationalized as trimester of infection, coded as a categorical exposure with four levels: first, second, or third trimester infection, and unexposed group as the reference group. Within this modeling framework, we first estimated third-trimester contrasts versus unexposed group to test the hypothesis that exposure in the third trimester causes greater neurodevelopmental risk than no exposure. Successively, we conducted linear contrasts comparing third-trimester infection with SARS-CoV-2 infection occurring in the first and second trimester (i.e., third trimester versus first and second trimesters combined) to test the hypothesis that exposure in the third trimester causes greater neurodevelopmental risk than exposure occurring earlier in pregnancy. Finally, we explored pairwise contrasts among all trimesters (i.e., third vs first, third vs second, and first vs second).

## 15.2 Results

No significant associations were observed when comparing third-trimester SARS-CoV-2 infection exposure to the unexposed group (Table S5) or to first and second trimesters combined (Table S6) in either minimally or fully adjusted models. Exploratory pairwise contrasts comparing all trimesters revealed nominally significantly lower SRS scores in the second-trimester exposure group compared to the third-trimester group (Table S7; Figure S3) in both minimally and fully adjusted models. These results did not survive FDR adjustment for multiple comparisons (q>0.05). Furthermore, stepwise adjustment suggested that this association was largely explained by sociodemographic differences between trimester groups, particularly the higher proportion of White mothers (race/ethnicity: 27.7% attenuation) and lower pre-pregnancy BMI (17.8% attenuation) in the second-trimester group (Figures S4–S6).

## Table S5. Results from contrasts between exposure to SARS-CoV-2 infection during the third trimester (n=66) and no exposure (n=557) for each neurodevelopmental outcome

|  | **Minimally adjusted** | | | | **Fully adjusted** | | | |
| --- | --- | --- | --- | --- | --- | --- | --- | --- |
| **Outcome** | Beta | 95% CI | p-value | q-value | Beta | 95% CI | p-value | q-value |
| **CBCL – Internalizing** | 0.12 | (-1.40, 1.64) | 0.87 | 0.99 | -0.22 | (-1.78, 1.34) | 0.78 | 0.99 |
| **CBCL – Externalizing** | -0.06 | (-1.86, 1.73) | 0.94 | 0.99 | -0.46 | (-2.24, 1.31) | 0.61 | 0.99 |
| **CBCL – Total Problems** | 0.15 | (-4.61, 4.92) | 0.95 | 0.99 | -1.04 | (-5.88, 3.79) | 0.67 | 0.99 |
| **SRS^a^** | 3.16 | (-1.57, 7.88) | 0.20 | 0.99 | 1.86 | (-2.94, 6.66) | 0.48 | 0.99 |

**Note:** Minimally adjusted models included: child sex, age at assessment, maternal education, race and ethnicity, maternal age at delivery, pandemic timing, and parity. Fully adjusted models additionally included pre-pregnancy BMI, maternal mental health history, and gestational age at birth. First and second trimester exposures were not included in this analysis.

^a^For SRS outcome, group sizes are Third trimester n=64, Unexposed n=548.

BMI = body mass index; CBCL = Child Behavior Checklist; SRS = Social Responsiveness Scale

## Table S6. Results from contrasts between exposure to SARS-CoV-2 infection in the third trimester (n=66) vs first and second trimesters combined (n=52) for each neurodevelopmental outcome

|  | **Minimally adjusted** | | | | **Fully adjusted** | | | |
| --- | --- | --- | --- | --- | --- | --- | --- | --- |
| **Outcome** | Beta | 95% CI | p-value | q-value | Beta | 95% CI | p-value | q-value |
| **CBCL – Internalizing** | -0.05 | (-2.87, 2.77) | 0.97 | 0.99 | -0.57 | (-3.42, 2.28) | 0.70 | 0.99 |
| **CBCL – Externalizing** | 0.33 | (-2.81, 3.46) | 0.84 | 0.99 | -0.27 | (-3.34, 2.79) | 0.86 | 0.99 |
| **CBCL – Total Problems** | -0.02 | (-8.89, 8.84) | 0.99 | 0.99 | -1.77 | (-10.60, 7.06) | 0.69 | 0.99 |
| **SRS^a^** | 4.64 | (-3.93, 13.21) | 0.29 | 0.99 | 2.39 | (-6.45, 11.23) | 0.59 | 0.99 |

**Note:** Minimally adjusted models included: child sex, age at assessment, maternal education, race and ethnicity, maternal age at delivery, pandemic timing, and parity. Fully adjusted models additionally included pre-pregnancy BMI, maternal mental health history, and gestational age at birth. The unexposed group was not included in this analysis.

^a^For SRS outcome, group sizes are: Third trimester n=64 and First and Second trimesters combined n=50.

BMI = body mass index; CBCL = Child Behavior Checklist; SRS = Social Responsiveness Scale

## Table S7. Pairwise contrasts comparing all trimesters of SARS-CoV-2 infection exposure for each neurodevelopmental outcome

|  |  | **Minimally adjusted** | | | | **Fully adjusted** | | | |
| --- | --- | --- | --- | --- | --- | --- | --- | --- | --- |
| **Trimester contrast** | **Outcome** | Beta | 95% CI | p-value | q-value | Beta | 95% CI | p-value | q-value |
| *Third*  *vs*  *First* | **CBCL – Internalizing** | -2.68 | (-8.39, 3.02) | 0.36 | 0.99 | -3.04 | (-8.64, 2.57) | 0.29 | 0.99 |
|  | **CBCL – Externalizing** | -1.29 | (-6.47, 3.89) | 0.62 | 0.99 | -1.65 | (-6.59, 3.29) | 0.51 | 0.99 |
|  | **CBCL – Total Problems** | -4.71 | (-21.50, 12.09) | 0.58 | 0.99 | -5.84 | (-22.14, 10.47) | 0.48 | 0.99 |
|  | **SRS^a^** | -1.30 | (-16.69, 14.09) | 0.87 | 0.99 | -2.45 | (-17.88, 12.98) | 0.76 | 0.99 |
|  |  | Beta | 95% CI | p-value | q-value | Beta | 95% CI | p-value | q-value |
| *Third*  *vs*  *Second* | **CBCL – Internalizing** | 0.42 | (-1.81, 2.65) | 0.71 | 0.99 | 0.10 | (-2.11, 2.31) | 0.93 | 0.99 |
|  | **CBCL – Externalizing** | 0.52 | (-2.34, 3.37) | 0.72 | 0.99 | 0.14 | (-2.69, 2.98) | 0.92 | 0.99 |
|  | **CBCL – Total Problems** | 0.99 | (-6.38, 8.36) | 0.79 | 0.99 | -0.13 | (-7.44, 7.17) | 0.97 | 0.99 |
|  | **SRS^a^** | 9.28 | (2.46, 16.10) | <0.01 | 0.31 | 7.66 | (0.98, 14.34) | 0.02 | 0.50 |
|  |  | Beta | 95% CI | p-value | q-value | Beta | 95% CI | p-value | q-value |
| *First*  *vs*  *Second* | **CBCL – Internalizing** | 3.10 | (-2.69, 8.89) | 0.29 | 0.99 | 3.13 | (-2.53, 8.80) | 0.28 | 0.99 |
|  | **CBCL – Externalizing** | 1.81 | (-3.59, 7.21) | 0.51 | 0.99 | 1.79 | (-3.40, 6.99) | 0.50 | 0.99 |
|  | **CBCL – Total Problems** | 5.70 | (-11.50, 22.89) | 0.52 | 0.99 | 5.70 | (-10.98, 22.38) | 0.50 | 0.99 |
|  | **SRS^a^** | 10.58 | (-4.85, 26.02) | 0.18 | 0.99 | 10.11 | (-5.34, 25.56) | 0.20 | 0.99 |

**Note:** Minimally adjusted models included: child sex, age at assessment, maternal education, race and ethnicity, maternal age at delivery, pandemic timing, and parity. Fully adjusted models additionally included pre-pregnancy BMI, maternal mental health history, and gestational age at birth. Unexposed group was not included in this analysis.

^a^For SRS outcome, group sizes were: First trimester n=17, Second trimester n=33, Third trimester n=64.

BMI = body mass index; CBCL = Child Behavior Checklist; SRS = Social Responsiveness Scale

## Figure S3. Forest plots of the associations between gestational timing of SARS-CoV-2 infection and neurodevelopmental outcomes

**Note:** Forest plots displaying beta coefficients (95% CIs) for all gestational timing analyses and for minimally (blue circles) and fully (purple triangles) adjusted models. For each neurodevelopmental outcome (CBCL Internalizing, CBCL Externalizing, CBCL Total Problems, and SRS), results are presented from top to bottom for the five hypothesized contrasts. From top to bottom for each plot, contrasts are: i) first versus second trimester, ii) third versus second trimester, iii) third versus first trimester, iv) third versus first and second trimesters combined, and v) third trimester versus unexposed. Minimally adjusted models included: child sex, age at assessment, maternal education, race and ethnicity, maternal age at delivery, pandemic timing, and parity. Fully adjusted models additionally included pre-pregnancy BMI, maternal mental health history, and gestational age at birth.

BMI = body mass index; CBCL = Child Behavior Checklist; SRS = Social Responsiveness Scale; *p<0.05

## Figure S4. Stepwise attenuation of the SARS-CoV-2 infection exposure in the third vs second trimester with SRS outcome

**Note:** Forest plot (left panel) displaying beta coefficients (95% CI) after sequential covariate addition ordered by largest absolute change. The table (right panel) displays absolute and step-specific percent attenuation. Race/ethnicity (27.7%) and pre-pregnancy body mass index (BMI; 17.8%) were the strongest attenuators. Remaining covariates contributed minimal change (<5% each).

BMI = body mass index; SRS = Social Responsiveness Scale

## Figure S5. Maternal pre-pregnancy BMI distribution in the second and third trimesters of gestational SARS-CoV-2 infection

**Note:** The boxes represent the IQR with the median value indicated by the horizontal line for the second (n=33; yellow) and third (n=64; blue) trimesters. Wilcoxon rank-sum test indicated that individuals infected during the second trimester had significantly lower pre-pregnancy body mass index (BMI) than those infected during the third trimester (p<0.01).

BMI = body mass index

## Figure S6. Maternal race/ethnicity distribution and effects on SRS score difference between second and third trimesters of gestational SARS-CoV-2 infection

|  | **Exposure timing** | | |
| --- | --- | --- | --- |
| **Race/ethnicity** | **Overall**  **(n=97)** | **Second trimester**  **(n=33)** | **Third trimester**  **(n=64)** |
| **White** | 31 (32.0%) | 15 (45.5%) | 16 (25.0%) |
| **Hispanic** | 37 (38.1%) | 10 (30.3%) | 27 (42.2%) |
| **Black** | 23 (23.7%) | 6 (18.2%) | 17 (26.6%) |
| **Asian** | 4 (4.1%) | 2 (6.1%) | 2 (3.1%) |
| **Other** | 2 (2.1%) | 0 (0.0%) | 2 (3.1%) |

**Note:** Figure: Beta coefficients and 95% confidence intervals are derived from a fully adjusted linear regression model with robust (HC3) standard errors including child sex, age at assessment, gestational age at birth, parity, maternal age at delivery, pre-pregnancy BMI, pandemic timing, education, and history of maternal mental illness. White mothers are used as the reference group because they constituted the largest group in the overall sample. Positive beta coefficients indicate higher SRS scores (greater social impairment) compared to White mothers; negative coefficients indicate lower scores. Table: Distribution (n, %) of maternal race/ethnicity for second and third trimesters of gestational SARS-CoV-2 infection.

BMI = body mass index

# Appendix S16. Sensitivity analysis excluding participants recruited from the Gen C-SF cohort

## 16.1 SARS-CoV-2 infection

### **Table S8.** Linear regression results (HC3 robust standard errors) results of the association between prenatal exposure to SARS-CoV-2 infection and neurodevelopmental outcomes excluding participants recruited from the Gen C-SF cohort

|  |  | **Minimally adjusted** | | | | **Fully adjusted** | | | |
| --- | --- | --- | --- | --- | --- | --- | --- | --- | --- |
| **Outcome** | **n** | Beta | 95% CI | p-value | q-value | Beta | 95% CI | p-value | q-value |
| **CBCL – Internalizing** | 634 | 0.16 | (-0.76, 1.07) | 0.70 | 0.70 | 0.01 | (-0.93, 0.89) | 0.98 | 0.99 |
| **CBCL – Externalizing** | 634 | -0.25 | (-1.43, 1.05) | 0.68 | 0.74 | -0.53 | (-1.76, 0.76) | 0.37 | 0.47 |
| **CBCL – Total Problems** | 634 | 0.14 | (-2.82, 3.33) | 0.92 | 0.92 | -0.47 | (-3.72, 2.65) | 0.74 | 0.74 |
| **SRS** | 621 | -0.24 | (-3.94, 4.08) | 0.89 | 0.89 | -0.86 | (-4.59, 3.13) | 0.63 | 0.67 |

**Note:** The sensitivity analysis excludes participants recruited from the Gen C-SF cohort (n=41) from the total sample, resulting in final group sizes of 533 unexposed and 101 exposed for CBCL and 524 unexposed and 97 exposed for SRS. Unexposed groups are the reference groups. Minimally adjusted models included: child sex, age at assessment, maternal education, race and ethnicity, maternal age at delivery, pandemic timing, and parity. Fully adjusted models additionally included pre-pregnancy BMI, maternal mental health history, and gestational age at birth. Statistical significance (FDR-adjusted q<0.05) is indicated in bold.

BMI = body mass index; CBCL = Child Behavior Checklist; SRS = Social Responsiveness Scale

### **Figure S7.** Sensitivity analysis: Forest plot of the association between prenatal exposure to SARS-CoV-2 infection and neurodevelopmental outcomes excluding participants recruited from the Gen C-SF cohort

**Note:** Minimally adjusted models included: child sex, age at assessment, maternal education, race and ethnicity, maternal age at delivery, pandemic timing, and parity. Fully adjusted models additionally included pre-pregnancy BMI, maternal mental health history, and gestational age at birth. Participants recruited from the Gen C-SF cohort (n=41) were excluded, resulting in final group sizes of 533 unexposed and 101 exposed for CBCL and 524 unexposed and 97 exposed for SRS. Data are presented as Beta coefficients and 95% confidence intervals, with the unexposed groups representing the reference groups.

BMI = body mass index; CBCL = Child Behavior Checklist; SRS = Social Responsiveness Scale

## 16.2 COVID-19 vaccination

### **Table S9.** Linear regression results (HC3 robust standard errors) results of the association between prenatal exposure to COVID-19 vaccination and neurodevelopmental outcomes excluding participants recruited from the Gen C-SF cohort

|  |  | **Minimally adjusted** | | | | **Fully adjusted** | | | |
| --- | --- | --- | --- | --- | --- | --- | --- | --- | --- |
| **Outcome** | **n** | Beta | 95% CI | p-value | q-value | Beta | 95% CI | p-value | q-value |
| **CBCL – Internalizing** | 634 | 0.02 | (-1.05, 1.18) | 0.97 | 0.97 | 0.27 | (-0.78, 1.42) | 0.62 | 0.62 |
| **CBCL – Externalizing** | 634 | 0.51 | (-1.13, 2.50) | 0.62 | 0.62 | 0.86 | (-0.76, 2.92) | 0.29 | 0.58 |
| **CBCL – Total Problems** | 634 | 0.28 | (-3.64, 4.85) | 0.88 | 0.97 | 1.20 | (-2.60, 5.76) | 0.54 | 0.97 |
| **SRS** | 621 | -4.73 | (-8.56, -0.60) | 0.04 | 0.17 | -4.24 | (-8.38, 0.15) | 0.07 | 0.29 |

**Note:** The sensitivity analysis excludes participants recruited from the Gen C-SF cohort (n=41) from the total sample, resulting in final group sizes of 579 unexposed and 55 exposed for CBCL and 566 unexposed and 55 exposed for SRS. Unexposed groups are the reference groups. Minimally adjusted models included: child sex, age at assessment, maternal education, race and ethnicity, maternal age at delivery, pandemic timing, and parity. Fully adjusted models additionally included pre-pregnancy BMI, maternal mental health history, and gestational age at birth.

BMI = body mass index; CBCL = Child Behavior Checklist; SRS = Social Responsiveness Scale

### **Figure S8.** Sensitivity analysis: Forest plot of the association between prenatal exposure to COVID-19 vaccination and neurodevelopmental outcomes excluding participants recruited from the Gen C-SF cohort

**Note:** Minimally adjusted models included: child sex, age at assessment, maternal education, race and ethnicity, maternal age at delivery, pandemic timing, and parity. Fully adjusted models additionally included pre-pregnancy BMI, maternal mental health history, and gestational age at birth. Participants recruited from the Gen C-SF cohort (n=41) were excluded, resulting in final group sizes of 579 unexposed and 55 exposed for CBCL and 566 unexposed and 55 exposed for SRS. Data are presented as Beta coefficients and 95% confidence intervals, with the unexposed groups representing the reference groups.

BMI = body mass index; CBCL = Child Behavior Checklist; SRS = Social Responsiveness Scale

## 16.3 Infection severity

### **Figure S9.** Sensitivity analysis: Forest plot of the association between SARS-CoV-2 infection severity and neurodevelopmental outcomes excluding participants recruited from the Gen C-SF cohort

**Note:** Forest plot displaying beta coefficients (95% confidence intervals) from multivariable Linear regression (HC3 robust standard errors) models comparing each SARS-CoV-2 infection severity group to the unexposed reference group (vertical dashed grey line at β=0). Participants recruited from the Gen C-SF cohort were excluded, resulting in final group sizes of Asymptomatic n=58, Mild n=32, Moderate/Severe n=11, Unexposed n=533 for CBCL and Asymptomatic n=56, Mild n=31, Moderate/Severe n=10, Unexposed n=524 for SRS. Fully adjusted model included: child sex, age at assessment, maternal education, race and ethnicity, maternal age at delivery, pandemic timing, parity, pre-pregnancy BMI, maternal mental health history, and gestational age at birth. Only results from fully adjusted models are reported.

BMI = body mass index; CBCL = Child Behavior Checklist; SRS = Social Responsiveness Scale

## 16.4 Infection timing

### **Figure S10.** Sensitivity analysis: Forest plots of the associations between gestational timing of SARS-CoV-2 infection and neurodevelopmental outcomes excluding participants recruited from the Gen C-SF cohort

**Note:** Forest plots displaying beta coefficients (95% CIs) for all gestational timing analyses. From top to bottom for each plot, contrasts are: i) third trimester versus unexposed, ii) third versus first and second trimesters combined, iii) third versus first trimester, iv) third versus second trimester, and v) first versus second trimester. Participants recruited from the Gen C-SF (n=41) cohort were excluded, resulting in final sample sizes of Unexposed n=533, First trimester exposure n=14, Second trimester exposure n=30, and Third trimester exposure n=57 for CBCL and Unexposed n=524, First trimester exposure n=14, Second trimester exposure n=28, and Third trimester exposure n=55 for SRS. Fully adjusted model included: child sex, age at assessment, maternal education, race and ethnicity, maternal age at delivery, pandemic timing, parity, pre-pregnancy BMI, maternal mental health history, and gestational age at birth. Only results from fully adjusted models are reported.

BMI = body mass index; CBCL = Child Behavior Checklist; SRS = Social Responsiveness Scale; *p<0.05

# Appendix S17. Primary analysis stratified by age category

## 17.1 Statistical analysis

To examine whether associations between prenatal exposures and child outcomes varied by age at assessment, we repeated the fully adjusted models for SARS-CoV-2 infection and COVID-19 vaccination within age groups, and tested exposure × age-group interactions in the full sample. For SARS-CoV-2 infection exposure, we used three age groups (24–36, 37–48, and ≥49 months). For vaccination, only 7 children in the 24–36 month group were vaccinated (probably due to the low availability of COVID-19 vaccines in the early stages of the pandemic), so this group was combined with the 37–48 month group, yielding two groups: 24–48 and ≥49 months. Children older than 60 months (n = 19) were included in the ≥49 month group. Fully adjusted model included: child sex, age at assessment, maternal education, race and ethnicity, maternal age at delivery, pandemic timing, parity, pre-pregnancy BMI, maternal mental health history, and gestational age at birth. Only results from fully adjusted models are reported. Models were estimated using linear regression with heteroscedasticity-consistent (HC3) standard errors and 95% confidence intervals and p-values were based on these standard errors, as in the main analyses. False discovery rate (FDR) correction (Benjamini-Hochberg) was applied across the four outcomes within each age group and within each interaction test.

## 17.2 Results

### 17.2.1 SARS-CoV-2 infection

Among 675 children with CBCL data, 200 were assessed at 24–36 months (18 exposed), 359 at 37–48 months (83 exposed), and 116 at ≥49 months (17 exposed); corresponding numbers for the 662 children with SRS data are given in Table S10. Prenatal infection was not associated with any outcome in any age group (all q>0.05), and there was no evidence that associations differed by age (interaction p and q>0.05) (Table S10).

### 17.2.2 COVID-19 vaccination

Among the 675 children with CBCL data, 576 were assessed at 24–48 months (56 exposed) and 99 at ≥49 months (12 exposed); corresponding numbers for the 662 children with SRS data are given in Table S11. Prenatal exposure to COVID-19 vaccination was not associated with any outcome in either age group (all q>0.05), and interaction tests were non-significant (all q>0.05) (Table S11).

### **Table S10.** Association between prenatal SARS-CoV-2 infection exposure and child neurodevelopmental outcomes stratified by age group at assessment

|  | **24–36 mo**  *Unexposed=182*  *Exposed=18* | | | **37–48 mo**  *Unexposed=276*  *Exposed=83* | | | **≥49 mo**  *Unexposed=99*  *Exposed=17* | | | **Interaction**  *Exposure x age* | |
| --- | --- | --- | --- | --- | --- | --- | --- | --- | --- | --- | --- |
| **Outcome** | **Beta (95% CI)** | **p-value** | **q-value** | **Beta (95% CI)** | **p-value** | **q-value** | **Beta (95% CI)** | ***p-value*** | ***q-value*** | ***p-value*** | ***q-value*** |
| **CBCL – Int.** | 0.92  (-2.49, 4.33) | 0.60 | 0.65 | 0.64  (-1.08, 2.36) | 0.46 | 0.97 | -0.78  (-4.14, 2.58) | 0.65 | 0.69 | 0.54 | 0.72 |
| **CBCL – Ext.** | 1.44  (-3.13, 6.01) | 0.54 | 0.65 | -0.03  (-1.82, 1.76) | 0.97 | 0.97 | -2.18  (-5.90, 1.54) | 0.25 | 0.69 | 0.41 | 0.72 |
| **CBCL – Tot.** | 3.70  (-8.41, 15.80) | 0.55 | 0.65 | 0.61  (-4.56, 5.78) | 0.82 | 0.93 | -2.93  (-12.96, 7.11) | 0.56 | 0.69 | 0.59 | 0.72 |
|  | **24–36 mo**  *Unexposed=180*  *Exposed=17* | | | **37–48 mo**  *Unexposed=274*  *Exposed=82* | | | **≥49 mo**  *Unexposed=94*  *Exposed=15* | | | **Interaction**  *Exposure x age* | |
| **SRS** | 2.68  (-8.86, 14.22) | 0.65 | 0.65 | -0.18  (-5.64, 5.27) | 0.95 | 0.97 | 2.53  (-9.86, 14.92) | 0.69 | 0.69 | 0.72 | 0.72 |

**Note:** Estimates are Beta coefficients (95% CI) from fully adjusted linear regression models with robust (HC3) standard errors comparing children prenatally exposed and unexposed to SARS-CoV-2 infection, fitted in the full sample and within each age group (24–36, 37–48, ≥49 months at assessment). Fully adjusted models included: child sex, age at assessment, maternal education, race and ethnicity, maternal age at delivery, pandemic timing, parity, pre-pregnancy BMI, maternal mental health history, and gestational age at birth. Interaction p-values are calculated from exposure x age-group terms. Interaction q-values are Benjamini-Hochberg false discovery rate-adjusted p-values across the four outcomes.

BMI = body mass index; CBCL = Child Behavior Checklist; Ext. = Externalizing; Int. = Internalizing; mo = months; SRS = Social Responsiveness Scale; Tot. = Total Problems

### **Table S11.** Association between prenatal COVID-19 vaccination exposure and child neurodevelopmental outcomes stratified by age group at assessment

|  | **24–48 mo**  *Unexposed=520*  *Exposed=56* | | | **≥49 mo**  *Unexposed=87*  *Exposed=12* | | | **Interaction**  *Exposure x age* | |
| --- | --- | --- | --- | --- | --- | --- | --- | --- |
| **Outcome** | **Beta (95% CI)** | **p-value** | **q-value** | **Beta (95% CI)** | **p-value** | **q-value** | ***p-value*** | ***q-value*** |
| **CBCL – Int.** | 0.03 (-1.31, 1.37) | 0.97 | 0.97 | -1.00 (-5.73, 3.73) | 0.67 | 0.71 | 0.52 | 0.52 |
| **CBCL – Ext.** | 0.68 (-1.07, 2.43) | 0.45 | 0.89 | 3.65 (-6.27, 13.58) | 0.47 | 0.71 | 0.32 | 0.52 |
| **CBCL – Tot.** | 0.82 (-3.59, 5.23) | 0.72 | 0.95 | 3.57 (-15.59, 22.73) | 0.71 | 0.71 | 0.33 | 0.52 |
|  | **24–48 mo**  *Unexposed=513*  *Exposed=56* | | | **≥49 mo**  *Unexposed=81*  *Exposed=12* | | | **Interaction**  *Exposure x age* | |
| **SRS** | -5.60 (-10.55, -0.65) | 0.03 | 0.11 | -8.56 (-24.72, 7.61) | 0.30 | 0.71 | 0.49 | 0.52 |

**Note:** Estimates are Beta coefficients (95% CI) from fully adjusted with robust (HC3) standard errors models comparing children prenatally exposed and unexposed to COVID-19 vaccination, fitted in the full sample and within each age group (24–48, ≥49 months at assessment). Fully adjusted models included: child sex, age at assessment, maternal education, race and ethnicity, maternal age at delivery, pandemic timing, parity, pre-pregnancy BMI, maternal mental health history, and gestational age at birth. Interaction p-values are calculated from exposure x age-group terms. Interaction q-values are Benjamini-Hochberg false discovery rate-adjusted p-values across the four outcomes.

BMI = body mass index; CBCL = Child Behavior Checklist; Ext. = Externalizing; Int. = Internalizing; mo = months; SRS = Social Responsiveness Scale; Tot. = Total Problems

# Appendix S18. Linear regression results (HC3 robust standard errors) for the association between prenatal COVID-19 vaccination and CBCL Internalizing outcomes including all covariates

|  |  | **Minimally adjusted** | | | | **Fully adjusted** | | | |
| --- | --- | --- | --- | --- | --- | --- | --- | --- | --- |
| **Variable** | **n** | Beta | 95% CI | p-value | q-value | Beta | 95% CI | p-value | q-value |
| **COVID-19 vaccination exposure:** Unexposed [ref] | 607 |  |  |  |  |  |  |  |  |
| Exposed | 68 | 0.23 | (-0.75, 1.28) | 0.65 | 0.65 | 0.41 | (-0.52, 1.44) | 0.42 | 0.45 |
| **Child sex:** Male [ref] | 342 |  |  |  |  |  |  |  |  |
| Female | 333 | -0.27 | (-0.81, 0.29) | 0.33 | 0.40 | -0.30 | (-0.85, 0.28) | 0.29 | 0.41 |
| **Parity:** Nulliparous [ref] | 338 |  |  |  |  |  |  |  |  |
| Multiparous | 337 | -0.53 | (-1.11, 0.04) | 0.08 | 0.14 | -0.45 | (-1.02, 0.12) | 0.14 | 0.30 |
| **Gestational age at birth** (days) | 675 | - | - | - | - | -0.01 | (-0.04, 0.02) | 0.25 | 0.41 |
| **Age at assessment** (months) | 675 | 0.04 | (0.01, 0.07) | <0.01 | **0.03** | 0.05 | (0.02, 0.08) | 0.001 | **<0.01** |
| **Maternal age at delivery** (years) | 675 | 0.02 | (-0.06, 0.09) | 0.63 | 0.65 | 0.00 | (-0.07, 0.07) | 0.96 | 0.96 |
| **Pre-pregnancy BMI** (kg/m^2^) | 675 | - | - | - | - | 0.04 | (-0.03, 0.10) | 0.11 | 0.28 |
| **Pandemic timing** (days) | 675 | 0.04 | (0.01, 0.08) | <0.01 | **0.03** | 0.02 | (-0.02, 0.06) | 0.34 | 0.41 |
| **History of maternal mental illness:** No [ref] | 413 |  |  |  |  |  |  |  |  |
| Yes | 262 | - | - | - | - | 1.23 | (0.51, 1.95) | <0.001 | **0.001** |
| **Race/ethnicity:** White [ref] | 293 |  |  |  |  |  |  |  |  |
| Hispanic | 178 | 0.76 | (-0.02, 1.57) | 0.06 | 0.12 | 0.53 | (-0.30, 1.43) | 0.20 | 0.38 |
| Black | 116 | 0.64 | (-0.26, 1.57) | 0.16 | 0.23 | 0.44 | (-0.52, 1.42) | 0.35 | 0.41 |
| Asian | 79 | 1.18 | (0.33, 2.16) | 0.01 | **0.03** | 1.30 | (0.46, 2.23) | <0.01 | **0.02** |
| Other^a^ | 9 | 3.34 | (-0.53, 15.70) | <0.01 | 0.03 | 3.35 | (-0.78, 15.89) | <0.01 | 0.02 |
| **Educational attainment:** Higher education [ref] | 464 |  |  |  |  |  |  |  |  |
| High school/Some college | 181 | 0.56 | (-0.31, 1.58) | 0.17 | 0.23 | 0.39 | (-0.49, 1.30) | 0.33 | 0.41 |
| No high school diploma | 30 | 2.96 | (0.50, 7.15) | <0.001 | **<0.001** | 2.72 | (0.22, 6.72) | <0.001 | **<0.01** |

**Note:** Minimally adjusted models included: child sex, age at assessment, maternal education, race and ethnicity, maternal age at delivery, pandemic timing, and parity. Fully adjusted models additionally included pre-pregnancy BMI, maternal mental health history, and gestational age at birth. For each covariate, the largest group served as the reference with Beta set to 0. Statistical significance (FDR-adjusted q<0.05) is indicated in bold.

^a^Due to the small sample size (n=9), the Race/ethnicity: Other category yielded an unstable and asymmetrical estimate, and this category was therefore treated as non-significant.

BMI = body mass index

# Appendix S19. Linear regression results (HC3 robust standard errors)for the association between prenatal COVID-19 vaccination and CBCL Externalizing outcomes including all covariates

|  |  | **Minimally adjusted** | | | | **Fully adjusted** | | | |
| --- | --- | --- | --- | --- | --- | --- | --- | --- | --- |
| **Variable** | **n** | Beta | 95% CI | p-value | q-value | Beta | 95% CI | p-value | q-value |
| **COVID-19 vaccination exposure:** Unexposed [ref] | 607 |  |  |  |  |  |  |  |  |
| Exposed | 68 | 0.92 | (-0.64, 2.58) | 0.22 | 0.29 | 1.42 | (-0.32, 2.95) | 0.13 | 0.27 |
| **Child sex:** Male [ref] | 342 |  |  |  |  |  |  |  |  |
| Female | 333 | -0.91 | (-1.69, -0.09) | 0.03 | 0.09 | -0.99 | (-1.78, -0.16) | 0.02 | 0.06 |
| **Parity:** Nulliparous [ref] | 338 |  |  |  |  |  |  |  |  |
| Multiparous | 337 | -0.53 | (-1.44, 0.32) | 0.24 | 0.29 | -0.41 | (-1.32, 0.41) | 0.36 | 0.49 |
| **Gestational age at birth** (days) | 675 | - | - | - | - | 0.00 | (-0.05, 0.05) | 0.96 | 0.96 |
| **Age at assessment** (months) | 675 | 0.05 | (0.00, 0.09) | 0.04 | 0.10 | 0.06 | (0.01, 0.10) | 0.01 | **0.05** |
| **Maternal age at delivery** (years) | 675 | -0.01 | (-0.10, 0.10) | 0.89 | 0.89 | -0.02 | (-0.12, 0.08) | 0.62 | 0.66 |
| **Pre-pregnancy BMI** (kg/m^2^) | 675 | - | - | - | - | 0.04 | (-0.04, 0.13) | 0.22 | 0.33 |
| **Pandemic timing** (days) | 675 | 0.06 | (0.01, 0.11) | 0.02 | 0.09 | 0.02 | (-0.04, 0.07) | 0.52 | 0.62 |
| **History of maternal mental illness:** No [ref] | 413 |  |  |  |  |  |  |  |  |
| Yes | 262 | - | - | - | - | 2.16 | (1.15, 3.18) | <0.001 | **<0.001** |
| **Race/ethnicity:** White [ref] | 293 |  |  |  |  |  |  |  |  |
| Hispanic | 178 | 0.93 | (-0.18, 2.20) | 0.12 | 0.20 | 0.81 | (-0.51, 2.15) | 0.19 | 0.32 |
| Black | 116 | 1.13 | (-0.13, 2.51) | 0.10 | 0.19 | 1.01 | (-0.45, 2.46) | 0.15 | 0.28 |
| Asian | 79 | 1.57 | (0.20, 3.12) | 0.02 | 0.09 | 1.88 | (0.59, 3.36) | <0.01 | **0.03** |
| Other^a^ | 9 | 2.82 | (-1.20, 12.45) | 0.13 | 0.20 | 3.37 | (-0.93, 11.99) | 0.07 | 0.17 |
| **Educational attainment:** Higher education [ref] | 464 |  |  |  |  |  |  |  |  |
| High school/Some college | 181 | 0.64 | (-0.56, 1.88) | 0.30 | 0.32 | 0.38 | (-0.80, 1.57) | 0.54 | 0.62 |
| No high school diploma | 30 | 4.57 | (1.17, 9.15) | <0.001 | **<0.001** | 4.33 | (0.88, 8.49) | <0.001 | **<0.001** |

**Note:** Minimally adjusted models included: child sex, age at assessment, maternal education, race and ethnicity, maternal age at delivery, pandemic timing, and parity. Fully adjusted models additionally included pre-pregnancy BMI, maternal mental health history, and gestational age at birth. For each covariate, the largest group served as the reference with Beta set to 0. Statistical significance (FDR-adjusted q<0.05) is indicated in bold.

^a^Due to the small sample size (n=9), the Race/ethnicity: Other category yielded an unstable and asymmetrical estimate, and this category was therefore treated as non-significant.

BMI = body mass index

# Appendix S20. Linear regression results (HC3 robust standard errors) for the association between prenatal COVID-19 vaccination and CBCL Total Problems outcomes including all covariates

|  |  | **Minimally adjusted** | | | | **Fully adjusted** | | | |
| --- | --- | --- | --- | --- | --- | --- | --- | --- | --- |
| **Variable** | **n** | Beta | 95% CI | p-value | q-value | Beta | 95% CI | p-value | q-value |
| **COVID-19 vaccination exposure:** Unexposed [ref] | 607 |  |  |  |  |  |  |  |  |
| Exposed | 68 | 1.55 | (-2.04, 5.33) | 0.39 | 0.43 | 2.13 | (-1.22, 6.15) | 0.23 | 0.31 |
| **Child sex:** Male [ref] | 342 |  |  |  |  |  |  |  |  |
| Female | 333 | -2.10 | (-4.09, -0.00) | 0.04 | 0.08 | -2.28 | (-4.27, -0.20) | 0.02 | 0.05 |
| **Parity:** Nulliparous [ref] | 338 |  |  |  |  |  |  |  |  |
| Multiparous | 337 | -1.87 | (-3.94, 0.10) | 0.08 | 0.13 | -1.53 | (-3.74, 0.46) | 0.15 | 0.29 |
| **Gestational age at birth** (days) | 675 | - | - | - | - | -0.04 | (-0.14, 0.08) | 0.38 | 0.44 |
| **Age at assessment** (months) | 675 | 0.14 | (0.03, 0.24) | 0.01 | **0.03** | 0.16 | (0.05, 0.26) | <0.01 | **0.01** |
| **Maternal age at delivery** (years) | 675 | -0.01 | (-0.25, 0.24) | 0.90 | 0.90 | -0.05 | (-0.29, 0.18) | 0.65 | 0.65 |
| **Pre-pregnancy BMI** (kg/m^2^) | 675 | - | - | - | - | 0.12 | (-0.09, 0.35) | 0.16 | 0.29 |
| **Pandemic timing** (days) | 675 | 0.18 | (0.07, 0.31) | <0.01 | **0.01** | 0.08 | (-0.05, 0.22) | 0.17 | 0.29 |
| **History of maternal mental illness:** No [ref] | 413 |  |  |  |  |  |  |  |  |
| Yes | 262 | - | - | - | - | 4.86 | (2.39, 7.53) | <0.001 | **<0.001** |
| **Race/ethnicity:** White [ref] | 293 |  |  |  |  |  |  |  |  |
| Hispanic | 178 | 2.52 | (-0.28, 5.44) | 0.08 | 0.13 | 1.88 | (-1.36, 5.28) | 0.20 | 0.30 |
| Black | 116 | 2.47 | (-0.91, 5.91) | 0.13 | 0.17 | 1.82 | (-1.70, 5.60) | 0.27 | 0.34 |
| Asian | 79 | 4.53 | (1.27, 8.34) | <0.01 | **0.03** | 5.13 | (2.02, 8.62) | <0.01 | **<0.01** |
| Other^a^ | 9 | 10.90 | (-2.21, 45.74) | 0.02 | 0.04 | 11.50 | (-2.80, 45.08) | <0.01 | 0.03 |
| **Educational attainment:** Higher education [ref] | 464 |  |  |  |  |  |  |  |  |
| High school/Some college | 181 | 1.59 | (-1.46, 4.83) | 0.28 | 0.33 | 1.07 | (-1.85, 4.14) | 0.46 | 0.49 |
| No high school diploma | 30 | 13.79 | (2.81, 26.25) | <0.001 | **<0.001** | 13.23 | (2.28, 25.20) | <0.001 | **<0.001** |

**Note:** Minimally adjusted models included: child sex, age at assessment, maternal education, race and ethnicity, maternal age at delivery, pandemic timing, and parity. Fully adjusted models additionally included pre-pregnancy BMI, maternal mental health history, and gestational age at birth. For each covariate, the largest group served as the reference with Beta set to 0. Statistical significance (FDR-adjusted q<0.05) is indicated in bold.

^a^Due to the small sample size (n=9), the Race/ethnicity: Other category yielded an unstable and asymmetrical estimate, and this category was therefore treated as non-significant.

BMI = body mass index

# Appendix S21. Linear regression results (HC3 robust standard errors) for the association between prenatal COVID-19 vaccination and SRS outcomes including all covariates

|  |  | **Minimally adjusted** | | | | **Fully adjusted** | | | |
| --- | --- | --- | --- | --- | --- | --- | --- | --- | --- |
| **Variable** | **n** | Beta | 95% CI | p-value | q-value | Beta | 95% CI | p-value | q-value |
| **COVID-19 vaccination exposure:** Unexposed [ref] | 594 |  |  |  |  |  |  |  |  |
| Exposed | 68 | -4.01 | (-8.03, 0.16) | 0.06 | 0.09 | -3.65 | (-7.93, 0.32) | 0.09 | 0.14 |
| **Child sex:** Male [ref] | 338 |  |  |  |  |  |  |  |  |
| Female | 324 | -5.27 | (-7.87, -2.97) | <0.001 | **<0.001** | -5.57 | (-8.06, -3.36) | <0.001 | **<0.001** |
| **Parity:** Nulliparous [ref] | 331 |  |  |  |  |  |  |  |  |
| Multiparous | 331 | -1.68 | (-4.44, 0.81) | 0.20 | 0.24 | -1.94 | (-4.66, 0.52) | 0.14 | 0.19 |
| **Gestational age at birth** (days) | 662 | - | - | - | - | 0.02 | (-0.11, 0.13) | 0.74 | 0.74 |
| **Age at assessment** (months) | 662 | -0.03 | (-0.17, 0.11) | 0.64 | 0.65 | -0.02 | (-0.16, 0.11) | 0.74 | 0.74 |
| **Maternal age at delivery** (years) | 662 | -0.06 | (-0.34, 0.24) | 0.65 | 0.65 | -0.12 | (-0.40, 0.18) | 0.41 | 0.47 |
| **Pre-pregnancy BMI** (kg/m^2^) | 662 | - | - | - | - | 0.48 | (0.24, 0.71) | <0.001 | **<0.001** |
| **Pandemic timing** (days) | 662 | 0.26 | (0.10, 0.41) | <0.001 | **0.001** | 0.22 | (0.05, 0.40) | <0.01 | **<0.01** |
| **History of maternal mental illness:** No [ref] | 403 |  |  |  |  |  |  |  |  |
| Yes | 259 | - | - | - | - | 1.34 | (-1.35, 4.41) | 0.33 | 0.41 |
| **Race/ethnicity:** White [ref] | 287 |  |  |  |  |  |  |  |  |
| Hispanic | 173 | 5.42 | (2.30, 9.32) | <0.01 | **<0.01** | 3.18 | (-0.36, 7.21) | 0.08 | 0.13 |
| Black | 115 | 6.77 | (3.01, 11.59) | <0.001 | **0.001** | 3.67 | (-0.82, 8.98) | 0.07 | 0.13 |
| Asian | 78 | 6.91 | (3.28, 10.94) | <0.001 | **0.001** | 7.14 | (3.52, 11.31) | <0.001 | **0.001** |
| Other^a^ | 9 | 18.16 | (1.40, 72.87) | <0.001 | 0.001 | 17.35 | (-1.34, 62.00) | 0.001 | <0.01 |
| **Educational attainment:** Higher education [ref] | 458 |  |  |  |  |  |  |  |  |
| High school/Some college | 175 | 9.15 | (5.10, 13.17) | <0.001 | **<0.001** | 8.17 | (4.22, 12.11) | <0.001 | **<0.001** |
| No high school diploma | 29 | 14.99 | (6.23, 25.86) | <0.001 | **<0.001** | 13.99 | (5.22, 24.81) | <0.001 | **<0.001** |

**Note:** Minimally adjusted models included: child sex, age at assessment, maternal education, race and ethnicity, maternal age at delivery, pandemic timing, and parity. Fully adjusted models additionally included pre-pregnancy BMI, maternal mental health history, and gestational age at birth. For each covariate, the largest group served as the reference with Beta set to 0. Statistical significance (FDR-adjusted q<0.05) is indicated in bold.

^a^Due to the small sample size (n=9), the Race/ethnicity: Other category yielded an unstable and asymmetrical estimate, and this category was therefore treated as non-significant.

BMI = body mass index

# Appendix S22. Sensitivity analysis: results from the association between prenatal exposure to COVID-19 vaccination and neurodevelopmental outcomes excluding participants vaccinated before pregnancy

|  |  |  | **Minimally adjusted** | | | | **Fully adjusted** | | | |
| --- | --- | --- | --- | --- | --- | --- | --- | --- | --- | --- |
| **Outcome** | **Unexposed** | **Exposed** | Beta | 95% CI | p-value | q-value | Beta | 95% CI | p-value | q-value |
| **CBCL – Internalizing** | n=555 | n=68 | 0.10 | (-0.89, 1.63) | 0.85 | 0.85 | 0.35 | (-0.69, 1.40) | 0.50 | 0.50 |
| **CBCL – Externalizing** | n=555 | n=68 | 0.59 | (-0.80, 2.29) | 0.46 | 0.85 | 0.82 | (-0.61, 2.61) | 0.29 | 0.50 |
| **CBCL – Total Problems** | n=555 | n=68 | 0.67 | (-2.73, 4.98) | 0.72 | 0.85 | 1.52 | (-2.04, 5.68) | 0.42 | 0.50 |
| **SRS** | n=542 | n=68 | -4.71 | (-8.83, -0.57) | 0.03 | 0.13 | -3.76 | (-7.98, 0.69) | 0.09 | 0.35 |

**Note:** Individuals vaccinated before pregnancy n=52. Minimally adjusted models included: child sex, age at assessment, maternal education, race and ethnicity, maternal age at delivery, pandemic timing, and parity. Fully adjusted models additionally included pre-pregnancy BMI, maternal mental health history, and gestational age at birth.

BMI = body mass index; CBCL = Child Behavior Checklist; SRS = Social Responsiveness Scale

# Appendix S23. Sensitivity analysis: Forest plot of the association between prenatal exposure to COVID-19 vaccination and neurodevelopmental outcomes excluding participants vaccinated before pregnancy

**Note:** Minimally adjusted models included: child sex, age at assessment, maternal education, race and ethnicity, maternal age at delivery, pandemic timing, and parity. Fully adjusted models additionally included pre-pregnancy BMI, maternal mental health history, and gestational age at birth. Individuals vaccinated before pregnancy (n=52) were excluded, resulting in final unexposed group sizes of n=555 for CBCL and n=542 for SRS. Data are presented as Beta coefficients and 95% confidence intervals.

BMI = body mass index; CBCL = Child Behavior Checklist; SRS = Social Responsiveness Scale
